# Successful or Uncomplicated Use of Drug‐Coated Balloon Versus Drug‐Eluting Stent Strategies for De Novo Culprit Lesions in Acute Coronary Syndromes: Insights from a Nationwide Registry in Japan

**DOI:** 10.1161/JAHA.124.038071

**Published:** 2025-05-29

**Authors:** Tomonori Takahashi, Kyohei Yamaji, Shun Kohsaka, Hideki Ishii, Yuichiro Mori, Yuetsu Kikuta, Tetsuzo Wakatsuki, Koji Yamaguchi, Daisuke Nishioka, Kenya Kusunose, Tetsuya Amano, Masataka Sata, Ken Kozuma, The J‐PCI Registry Investigators, Shiro Uemura, Yutaka Hikichi, Osamu Iida, Yuji Ikari, Koichi Kaikita, Yoshio Kobayashi, Toshiro Shinke, Shinjo Sonoda, Saeko Takahashi, Kiyoshi Hibi, Yuichiro Maekawa, Shinichiro Yamada, Tetsuya Amano, Shun Kohsaka, Osamu Iida, Hideki Ishii, Kenichi Sakakura, Toshiro Shinke, Masato Nakamura, Tetsuya Matoba, Shun Kohsaka, Hideki Ishii, Kyohei Yamaji, Hirohiko Ando, Ayako Kunimura, Kenichi Sakakura, Takahiro Suzuki, Yuichiro Mori, Wada Hideki, Shun Kohsaka, Hideki Ishii, Kazuyuki Ozaki, Yuetsu Kikuta, Kunii Hiroyuki, Syoichi Kuramitsu, Mitsuaki Sawano, Jun Takahashi, Toshiharu Takeuchi, Yohei Numasawa, Tetsuya Matoba, Kyohei Yamaji, Tetsu Watanabe

**Affiliations:** ^1^ Department of Cardiovascular Medicine Tokushima University Hospital Tokushima Japan; ^2^ Department of Cardiovascular Medicine Kyoto University Kyoto Japan; ^3^ Department of Cardiology Keio University School of Medicine Tokyo Japan; ^4^ Department of Cardiovascular Medicine Gunma University Graduate School of Medicine Maebashi Japan; ^5^ Department of Human Health Sciences, Graduate School of Medicine Kyoto University Kyoto Japan; ^6^ Department of Cardiovascular Medicine Fukuyama Cardiovascular Hospital Hiroshima Japan; ^7^ Department of Medical Statistics, Research & Development Center Osaka Medical and Pharmaceutical University Osaka Japan; ^8^ Department of Cardiovascular Medicine, Nephrology, and Neurology, Graduate School of Medicine University of the Ryukyus Okinawa Japan; ^9^ Department of Cardiology Aichi Medical University Nagakute Japan; ^10^ Division of Cardiology Teikyo University Hospital Tokyo Japan

**Keywords:** non‐ST‐elevation acute coronary syndrome, paclitaxel‐coated balloon, percutaneous coronary intervention, real‐world data, ST‐elevation myocardial infarction, Catheter-Based Coronary and Valvular Interventions, Clinical Studies

## Abstract

**Background:**

Randomized trials demonstrated that drug‐coated balloon (DCB) was not inferior to drug‐eluting stent (DES) for acute coronary syndrome (ACS). However, generalizability in clinical settings remains unclear. The present study compared the outcomes of DCB and DES strategies in percutaneous coronary intervention for ACS within a nationwide procedure‐based registry.

**Methods and Results:**

This was a retrospective analysis of a cohort study from a prospective, nationwide registry between January 2017 and December 2020 in Japan, focusing on patients with ACS who underwent DCB or DES for a single de novo lesion. Patients who required bailout stenting after treatment with DCB were excluded from the analysis. The 1‐year incidence of all‐cause mortality, cardiovascular death, noncardiovascular death, nonfatal ACS, stroke, and major bleeding events was compared. A subgroup analysis included lesion‐based and ST‐elevation myocardial infarction/non‐ST‐elevation ACS stratifications. Among 5212 propensity score–matched patients with ACS, no significant differences were observed in the 1‐year incidence of all‐cause mortality (4.5% versus 4.6%, hazard ratio [HR], 0.92 [95% CI, 0.72–1.19]); cardiovascular death (2.5% versus 2.5%, HR, 0.90 [95% CI, 0.64–1.26]); noncardiovascular death (2.0% versus 2.1%, HR, 0.96 [95% CI, 0.65–1.42]); or nonfatal ACS (1.7% versus 2.0%, HR, 1.04 [95% CI, 0.70–1.54]) between DCB and DES. DCB was associated with a higher incidence of stroke (0.8% versus 0.3%, HR, 2.33 [95% CI, 1.06–5.08]) and lower incidence of major bleeding events (1.4% versus 2.3%, HR, 0.65 [95% CI, 0.43–0.99]); however, these results were not reproduced in the subgroup analysis.

**Conclusions:**

The DCB strategy for successfully treated ACS cases achieved similar clinical outcomes to DES after 1 year. Further studies with an extended follow‐up are needed to confirm these results.

**Nonstandard Abbreviations and Acronyms**
BMS: bare metal stent
DCB: drug‐coated balloon
DES: drug‐eluting stent
PS: propensity score

**Clinical Perspective**

**What Is New?**

- In a large Japanese nationwide registry, 1‐year clinical outcomes were similar between the successful implementation of the drug‐coated balloon strategy and the conventional drug‐eluting stent strategy for percutaneous coronary intervention in acute coronary syndrome.

**What Are the Clinical Implications?**

- Our findings expand the understanding of the drug‐coated balloon strategy in patients with acute coronary syndrome and may be helpful in selecting strategies for percutaneous coronary intervention in real‐world clinical practice.

Current guidelines advocate the use of drug‐eluting stents (DES) rather than balloon angioplasty or bare metal stents (BMS) in percutaneous coronary intervention (PCI) for acute coronary syndrome (ACS).^1^ However, the implantation of stents is inevitably associated with the risk of stent‐related complications, such as stent thrombosis and vascular endothelial dysfunction in culprit lesions.^2^, ^3^, ^4^, ^5^ Furthermore, while the incidence of stent thrombosis with new‐generation DES is low at 0.9% over 1 to 5 years, target lesion failure remains at 7.7% and continues to rise annually without reaching a plateau.

To mitigate these complications, an alternative strategy that eliminates the need for stent placement has been examined. An approach using a drug‐coated balloon (DCB) has been intensively investigated in recent years. The efficacy of this stent‐less approach using DCB for de novo lesions was demonstrated in several high‐quality randomized controlled trials.^6^, ^7^, ^8^, ^9^ In ACS, investigations into target lesion failure at 9 months among patients with non–ST‐segment–elevation myocardial infarction (the PEPCAD NSTMI trial)^10^ and the fractional flow reserve of infarct‐related lesions at 9 months in patients with ST‐elevation myocardial infarction (STEMI) both showed that the DCB strategy was not inferior to stent‐based interventions.^11^

While the body of evidence from randomized controlled trials is expanding, there is still a lack of large‐scale real‐world data on the “leaving nothing behind” strategy in the context of PCI for ACS, and debate surrounds the generalization of findings from randomized controlled trials. Therefore, we herein compared the clinical outcomes of PCI strategies using DCB versus DES in patients with ACS within a nationwide PCI registry in Japan.

## METHODS

The data, analytical methods, and study materials will not be publicly accessible to other researchers for the purpose of reproducing the results or replicating the methodology.

### Data Source

This study was a multicenter, nonrandomized analysis of patients with ACS in Japan, utilizing data from the J‐PCI OUTCOME (Japanese Percutaneous Coronary Intervention Outcome) Registry. Established and endorsed by the Japanese Association of Cardiovascular Intervention and Therapeutics, the J‐PCI OUTCOME Registry is a prospective, nationwide multicenter registry database that is designed to collect information on clinical outcomes 1 year after discharge in patients who have undergone PCI for coronary artery disease. An overview of and details on the J‐PCI OUTCOME Registry have already been reported^12^ and are also provided in Data S1. In brief, patient data were collected through chart reviews or telephone interviews from 172 representative PCI facilities across 47 prefectures (18% of PCI facilities in Japan), which met the selection criteria of an annual PCI case volume of [mt]200 cases at each standard performance facility. Data were then reviewed by responsible data managers at each facility and registered through the secure web‐based electronic data capture system managed by the National Clinical Database. Japanese Association of Cardiovascular Intervention and Therapeutics also conducts ≈20 site visits annually at randomly selected hospitals to ensure the integrity and completeness of data.

The protocol for J‐PCI OUTCOME registration received approval from the independent Central Ethics Committee of the Japan Clinical Research Promotion Network and Institutional Review Boards in each region. Patient consent for participation in the J‐PCI OUTCOME Registry was obtained through an opt‐out notification via the website or posters. This study adhered to the principles of the Declaration of Helsinki.

### Study Population

This analysis focused on patients who underwent PCI for ACS at JPCI OUTCOME participating facilities between January 2017 and December 2020 and were treated for a single de novo culprit lesion. In addition to baseline clinical characteristics and procedural data at the time of PCI, we evaluated 1‐year clinical outcomes.

The J‐PCI OUTCOME Registry included 165 266 patients who underwent PCI during the study period. Patients with diagnoses other than ACS, cases of ACS with restenotic or graft lesions as the culprit lesions, and the use of devices other than DCB or DES (eg, balloon angioplasty alone, BMS, or a bioresorbable scaffold) were excluded. In addition, due to the challenges associated with identifying the culprit lesion in cases in which multiple lesions were treated, these cases were excluded. Similarly, cases in which DCB and DES were used concurrently were excluded because it was not possible to reliably distinguish between a bailout strategy and hybrid strategy involving DCB and DES.

**Figure 1:**
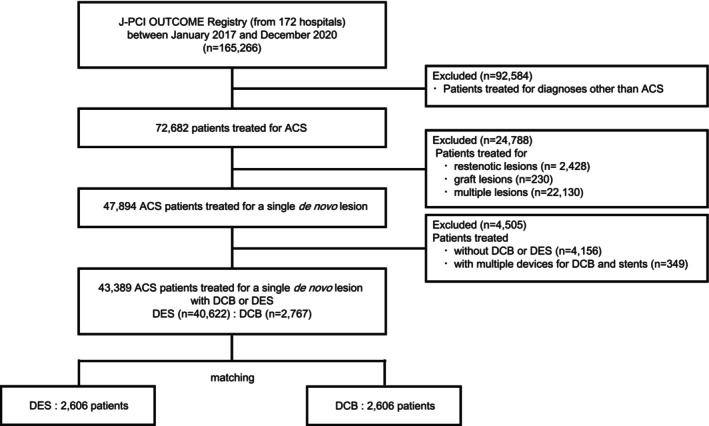
Flowchart of this study. ACS indicates acute coronary syndrome; DCB, drug‐coated balloon; and DES, drug‐eluting stent.

After the exclusion of these cases, propensity score (PS) matching was performed on a cohort comprising 40 622 patients following the DES strategy and 2767 following the DCB strategy (Figure 1). One‐year clinical outcomes were assessed, encompassing the incidence of all‐cause mortality, cardiovascular death, noncardiovascular death, nonfatal ACS, stroke, and major bleeding events. During the inclusion period, the only DCB approved in Japan was the SeQuent Please. Furthermore, only antiplatelet agents approved in Japan were analyzed.

### Sample Matching

To address potential confounding effects arising from differences in patient backgrounds, PS matching was used. Matching was performed within each group of the previously mentioned cohort, resulting in the selection of 2606 individuals from both the DCB and DES groups, totaling 5212 individuals for subsequent analyses. A subgroup analysis was conducted, categorizing the initial cohort based on the localization of the culprit lesion into 2 groups: major and minor lesion cohorts. Each cohort was defined according to the standard American Heart Association segmentation^13^: the major lesion cohort comprised segments 1, 2, 3, 5, 6, 7, 11, and 13, while the minor lesion cohort included all other segments (Figure S1). Matching was conducted within each group, and outcomes were evaluated. Furthermore, for the purpose of assessing outcomes based on the clinical classification of ACS, the initial cohort was further classified into 4 cohorts: STEMI in the major lesion cohort, non–ST‐elevation ACS in the major lesion cohort, STEMI in the minor lesion cohort, and non–ST‐elevation ACS in the minor lesion cohort. Matching was performed within each of these cohorts, followed by subsequent analyses.

### Definitions and Clinical Outcomes

Data definitions align with those established by the nationwide prospective multicenter database, the J‐PCI Registry, previously established by the Japanese Association of Cardiovascular Intervention and Therapeutics. Definitions for each clinical category have already been reported. Clinical events within 1 year post‐PCI were defined according to the 2017 Cardiovascular and Stroke Endpoint Definitions for Clinical Trials.^14^ Briefly, all‐cause mortality was defined as death from any cause. Cardiovascular death was defined to include sudden cardiac death and death due to ACS, heart failure, stroke, procedural complications, and bleeding. ACS encompassed STEMI, non–ST‐segment–elevation myocardial infarction, and unstable angina regardless of revascularization. Acute myocardial infarction was defined as an episode with persistent symptoms of myocardial ischemia and elevated cardiac markers. Nonfatal ACS was defined as including only spontaneous ACS and excluding periprocedural MI. Stroke was defined as an acute episode of localized or global neurological impairment due to vascular damage in the brain, spinal cord, or retina. Major bleeding events were defined as bleeding events requiring rehospitalization. Time‐to‐event was defined as days from discharge to the end point of tracking, which was either the outcome event or a 1‐year follow‐up. Definitions for other data are presented in Table S1.

### Statistical Analysis

Continuous variables are presented as the mean±SD and percentages (%) for categorical variables. To address differences in patient characteristics between the 2 strategies, PS matching was used. PS, defined as the probability of receiving the DCB strategy given the measured covariates, was estimated through a logistic regression model incorporating 11 clinically relevant covariates: age, sex, a history of PCI, a history of heart failure, a history of myocardial infarction, hypertension, diabetes, dyslipidemia, chronic obstructive pulmonary disease, dialysis, and a history of smoking. These covariates were selected based on their potential relationship with risk factors for outcomes, including mortality. PS matching was implemented with nearest neighbor 1:1 matching. The balance of each covariate between the 2 strategies before and after matching was assessed using standardized differences. An imbalance was considered to be small if the absolute value of the standardized difference was [lt]0.1.

Outcome comparisons between DCB and DES were conducted using Pearson's χ^2^ test and Cox proportional hazards models, expressing results as hazard ratios with the 95% CI. All statistical tests were 2‐sided, with a *P* value [lt] 0.05 indicating a significant difference. All statistical calculations and analyses were performed using R software version 4.3.2 (R Foundation for Statistical Computing, Vienna, Austria).

## RESULTS

### Patients and Procedural Backgrounds

**Figure 2:**
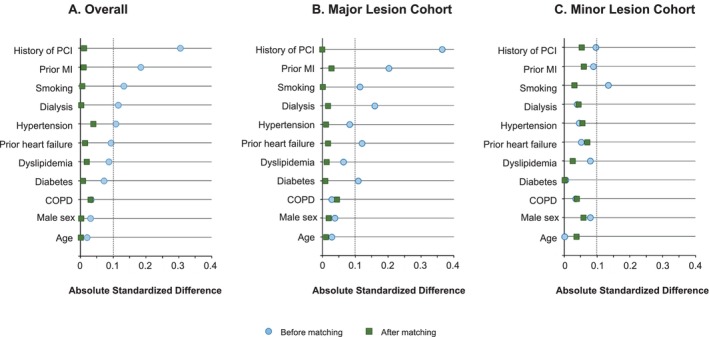
Love plot of selected variables. Love plot of selected variables in the (**A**) overall cohort, (**B**) major lesion cohort, and (**C**) minor lesion cohort. COPD indicates chronic obstructive pulmonary disease; MI, myocardial infarction; and PCI, percutaneous coronary intervention.

Before PS matching (n=43 389), the average age of patients was 69±13 years, with 76% being male. Table 1 and Table S2 show baseline characteristics and procedural backgrounds before and after PS matching. Before PS matching, patients undergoing the DCB strategy had a significantly higher prevalence of hypertension, diabetes, dyslipidemia, chronic kidney disease, dialysis, revascularization history, and history of MI than those treated with the DES strategy. Moreover, in the preoperative clinical presentation, STEMI cases were more prevalent in the DES strategy. The incidence of concomitant cardiogenic shock within 24 hours was higher in patients undergoing the DES strategy. Additionally, the use of antiplatelet medication revealed a higher prevalence of prasugrel in the DES strategy and a higher prevalence of clopidogrel in the DCB strategy. Regarding the devices used during PCI, rotational atherectomy was more frequently utilized in the DCB strategy, whereas thrombus aspiration and distal protection devices were more commonly used in the DES strategy. After PS matching, 5212 cases were included in the analysis. Covariate characteristics were well balanced between the matched sets (Figure 2), and the consistency of PS density matched after the matching process (Figure S2). In the matched cohort, the incidence of preprocedural cardiogenic shock was higher with the DES strategy than with the DCB strategy, and the former also included a higher percentage of patients with STEMI. Additionally, even after PS matching, rotational atherectomy remained more frequently utilized in the DCB strategy, whereas thrombus aspiration and distal protection devices continued to be more commonly used in the DES strategy. Baseline characteristics and procedural backgrounds in the subgroup analysis are shown in Tables S3 to S8.

**Table 1:** Baseline Characteristics Before and After Propensity Score Matching

	Nonmatching			Matching	
	All (n=43 389)	DES (n=40 622)	DCB (n=2767)	DES (n=2606)	DCB (n=2606)
Clinical characteristics					
Age, y	69±13	69±13	69±13	69±13	69±13
Male sex, n (%)	33 126 (76.3)	31 045 (76.4)	2081 (75.2)	1963 (75.3)	1967 (75.5)
History of PCI, n (%)	5752 (13.3)	5037 (12.4)	715 (25.9)	668 (25.6)	682 (26.2)
History of CABG, n (%)	611 (1.4)	526 (1.3)	85 (3.1)	61 (2.3)	82 (3.2)
Prior myocardial infarction, n (%)	5752 (13.3)	5037 (12.4)	715 (25.9)	392 (15.0)	402 (15.4)
Prior heart failure, n (%)	2715 (6.3)	2477 (6.2)	238 (8.7)	220 (8.4)	231 (8.9)
Hypertension, n (%)	30 228 (73.6)	28 173 (73.3)	2055 (77.8)	2072 (79.5)	2028 (77.8)
Diabetes, n (%)	15 133 (36.8)	14 074 (36.6)	1059 (40.1)	1055 (40.5)	1044 (40.1)
Dyslipidemia, n (%)	26 246 (63.9)	24 466 (63.6)	1780 (67.4)	1791 (68.7)	1767 (67.8)
Smoking, n (%)	16 692 (40.6)	15 769 (41.0)	923 (34.9)	897 (34.4)	905 (34.7)
Chronic kidney disease, n (%)	7361 (17.9)	6826 (17.8)	535 (20.3)	531 (20.4)	518 (19.9)
Dialysis, n (%)	1349 (3.3)	1193 (3.1)	156 (5.9)	149 (5.7)	151 (5.8)
Chronic obstructive lung disease, n (%)	1073 (2.6)	1016 (2.6)	57 (2.2)	44 (1.7)	56 (2.2)
Peripheral artery disease, n (%)	1607 (3.9)	1482 (3.9)	125 (4.7)	135 (5.2)	124 (4.8)
Baseline hemoglobin (g/dL)	13.8±2.1	13.8±2.1	13.5±2.2	13.6±2.2	13.5±2.2
Clinical presentations, n (%)					
ST‐elevation myocardial infarction	23 543 (54.3)	22 501 (55.4)	1042 (37.7)	1320 (50.7)	979 (37.6)
Cardiopulmonary arrest within 24 h	1637 (3.8)	1562 (3.9)	75 (2.7)	72 (2.8)	69 (2.7)
Cardiogenic shock within 24 h	2774 (6.4)	2679 (6.6)	95 (3.5)	159 (6.1)	90 (3.5)
Acute heart failure within 24 h	2918 (6.8)	2796 (6.9)	122 (4.4)	175 (6.7)	111 (4.3)
Number of diseased vessels					
1	29 430 (67.8)	27 526 (67.8)	1904 (68.8)	1725 (66.1)	1787 (68.6)
2	9489 (21.9)	8918 (22.0)	571 (20.6)	582 (22.3)	539 (20.7)
3	4470 (10.3)	4178 (10.3)	292 (10.6)	299 (11.5)	280 (10.7)
Left main disease	929 (2.1)	889 (2.2)	40 (1.4)	67 (2.6)	38 (1.5)
Preprocedural medications, n (%)					
Antiplatelets	37 576 (86.6)	35 199 (86.7)	2377 (85.9)	2331 (89.4)	2246 (86.2)
Aspirin	36 711 (97.5)	34 404 (84.7)	2307 (83.4)	2258 (86.6)	2182 (83.7)
Clopidogrel	6375 (16.9)	5829 (14.3)	546 (19.7)	475 (18.2)	512 (19.6)
Prasugrel	28 311 (75.2)	26 715 (65.8)	1596 (57.7)	1665 (63.9)	1518 (58.3)
Ticagrelor	41 (0.1)	39 (0.1)	2 (0.1)	4 (0.2)	2 (0.1)
Oral anticoagulants	1824 (4.2)	1693 (4.2)	131 (4.7)	119 (4.6)	124 (4.8)
Warfarin	667 (35.2)	616 (1.5)	51 (1.8)	50 (1.9)	49 (1.9)
Direct oral anticoagulant	1248 (2.9)	1166 (2.8)	82 (3.0)	74 (2.8)	77 (3.0)
Preprocedural mechanical circulatory assist device, n (%)					
ECMO	200 (0.5)	196 (0.5)	4 (0.1)	10 (0.4)	4 (0.2)
Impella	59 (0.1)	59 (0.1)	0 (0)	5 (0.2)	0 (0.0)
IABP	607 (1.4)	586 (1.4)	21 (0.8)	44 (1.7)	20 (0.8)
Lesion locations, n (%)					
Right coronary artery					
Segment 1	4970 (11.5)	4817 (11.9)	153 (5.5)	318 (12.2)	144 (5.5)
Segment 2	5078 (11.7)	4899 (12.1)	179 (6.5)	329 (12.6)	162 (6.2)
Segment 3	3848 (8.9)	3688 (9.1)	160 (5.8)	275 (10.6)	151 (5.8)
Segment 4	1087 (2.5)	871 (2.1)	216 (7.8)	75 (2.9)	208 (8.0)
Left main trunk	838 (1.9)	819 (2.0)	19 (0.7)	67 (2.6)	17 (0.7)
Left anterior descending artery					
Segment 6	10 704 (24.7)	10 285 (25.3)	419 (15.1)	581 (22.3)	388 (14.9)
Segment 7	8437 (19.4)	8116 (20.0)	321 (11.6)	510 (19.6)	301 (11.6)
Segment 8	253 (0.6)	205 (0.5)	48 (1.7)	17 (0.7)	47 (1.8)
Segment 9	1158 (2.7)	703 (1.7)	455 (16.4)	42 (1.6)	428 (16.4)
Segment 10	94 (0.2)	51 (0.1)	43 (1.6)	1 (0.0)	43 (1.7)
Left circumflex artery					
Segment 11	1926 (4.4)	1746 (4.3)	180 (6.5)	115 (4.4)	168 (6.5)
Segment 12 or HL	1085 (2.5)	885 (2.2)	200 (7.2)	50 (1.9)	189 (7.2)
Segment 13	2980 (6.9)	2763 (6.8)	217 (7.8)	179 (6.9)	206 (7.9)
Segment 14	582 (1.3)	480 (1.2)	102 (3.7)	31 (1.2)	100 (3.8)
Segment 15	349 (0.8)	294 (0.7)	55 (2.0)	16 (0.6)	54 (2.1)
Devices used during PCI, n (%)					
Rotational atherectomy	577 (1.3)	484 (1.2)	93 (3.4)	40 (1.5)	88 (3.4)
Thrombus aspiration	15 221 (35.1)	14 613 (36.0)	608 (22.0)	826 (31.7)	578 (22.2)
Distal protection device	2780 (6.4)	2736 (6.7)	44 (1.6)	152 (5.8)	42 (1.6)

Data are presented as n (%) or means ±SDs. CABG indicates coronary artery bypass graft; DCB, drug‐coated balloon; DES, drug‐eluting stent; ECMO, extracorporeal membrane oxygenation; HL, high lateral branch; IABP, intra‐aortic balloon pumping; and PCI, percutaneous coronary intervention.

### The Culprit Lesion and Periprocedural Complications

Before PS matching, the percentage of patients undergoing the DES strategy for major lesions was higher than that for the DCB strategy. In contrast, a slightly higher percentage of patients underwent the DCB strategy for minor lesions (Table 1, Table S2). Across all cohorts, including those with minor lesions, a slightly higher percentage of diagonal branches were treated with the DCB strategy, even after matching (Table 1, Tables S2, S4, S6, and S8). The incidence of periprocedural complications did not significantly differ between the 2 strategies after the exclusion of cases requiring bailout stenting in the DCB group (Table S9).

### Clinical Outcomes

**Figure 3:**
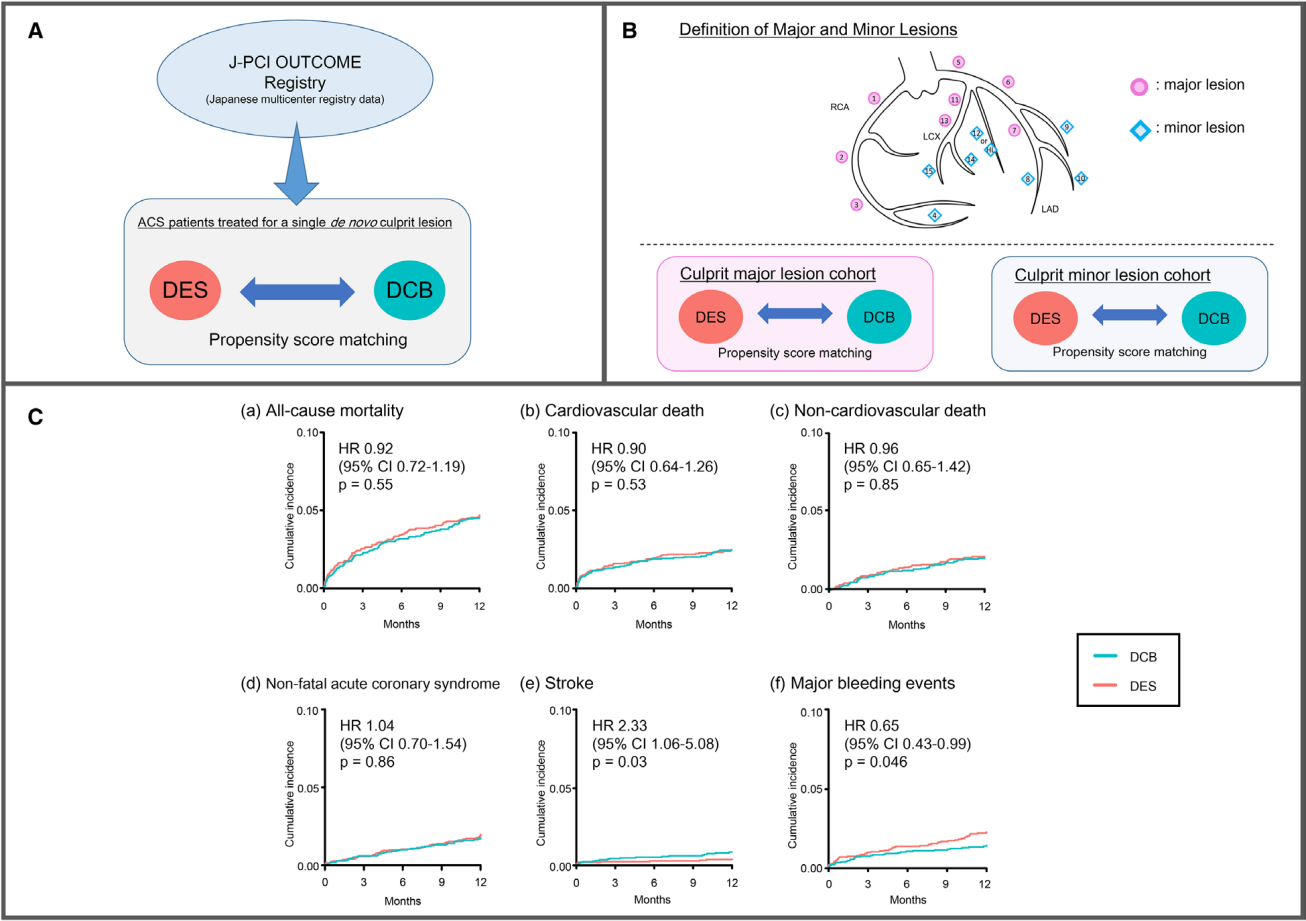
Study design and 1‐year outcomes of DCB vs DES strategies in acute coronary syndrome. **A**, Study overview. (**B**) Outline of the substudy. The cohort was divided into 2 groups based on the localization of the culprit lesion (upper panel), and propensity score matching was performed for each group (lower panel). (**C**) Comparison of 1‐y outcomes of the DCB vs DES strategies in patients with ACS. Kaplan–Meier curves show the cumulative incidence of (**a**) all‐cause mortality, (**b**) cardiovascular death, (**c**) noncardiovascular death, (**d**) nonfatal ACS, (**e**) stroke, and (**f**) major bleeding events for the DCB and DES strategies. Each HR represents the risk of events in the DCB strategy relative to the DES strategy. ACS indicates acute coronary syndrome; DCB, drug‐coated balloon; DES, drug‐eluting stent; HR, hazard ratio; LAD, left anterior descending artery; LCX, left circumflex artery and RCA, right coronary artery.

After PS matching, 1‐year clinical outcomes were similar between the DCB and DES strategies, with no significant differences in the incidence of all‐cause mortality (4.1% versus 4.3%, *P*=0.73), cardiovascular death (2.3% versus 2.3%, *P*=0.85), noncardiovascular death (1.8% versus 1.9%, *P*=0.76), or nonfatal ACS (4.1% versus 4.3%, *P*=0.73). Stroke was more frequent with the DCB strategy (0.7% versus 0.3%, *P*=0.03), while major bleeding events were less common with the DCB strategy (1.3% versus 2.0%, *P*=0.04). The 1‐year cumulative incidence of various outcomes between each strategy is shown in Figure 3.

### Outcomes Based on the Culprit Lesion and ACS Subtypes

**Figure 4:**
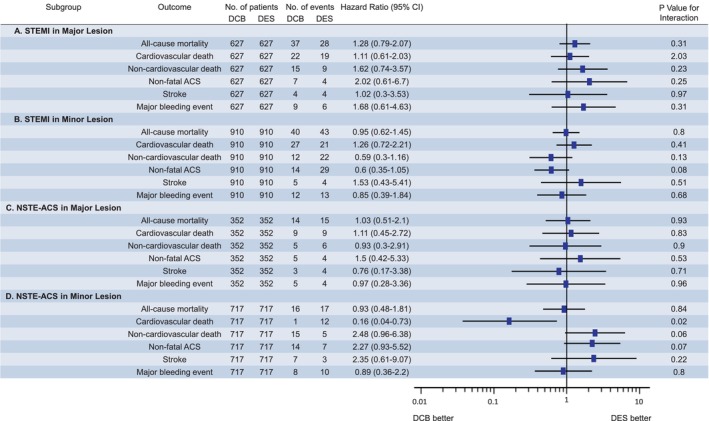
Subgroup analysis. The incidence of all‐cause mortality, cardiovascular death, noncardiovascular death, nonfatal ACS, stroke, and major bleeding events were compared between the DCB and DES strategies within each of the 4 subgroups: **(A)** STEMI in the major lesion cohort, **(B)** NSTE‐ACS in the major lesion cohort, **(C)** STEMI in the minor lesion cohort, and **(D)** NSTE‐ACS in the minor lesion cohort. ACS indicates acute coronary syndrome; DCB, drug‐coated balloon; DES, drug‐eluting stent; NSTE‐ACS, non–ST‐segment–elevation acute coronary syndrome; and STEMI, ST‐elevation myocardial infarction.

We compiled 1‐year follow‐up outcomes based on the culprit lesion and ACS subtypes in Table 2 and Figure 4. In the major and minor lesion cohorts, no significant differences were observed in the incidence of each outcome between the DCB and DES strategies. In the analysis accounting for ACS subtypes, among those with non–ST‐elevation ACS in the minor lesion cohort, patients who underwent the DCB strategy had a lower incidence of cardiac death at 1 year than those treated with DES. However, in the other subgroups, no significant differences were noted in the incidence of each outcome between the 2 strategies.

**Table 2:** One‐Year Outcomes for Propensity Score Matching

Outcomes	Major lesion cohort			*P* value	Minor lesion cohort			*P* value
	DCB (n=1533)	DES (n=1534)	HR (95% CI)		DCB (n=1052)	DES (n=1065)	HR (95% CI)	
All‐cause mortality	77 (5.4)	71 (5.0)	1.05 (0.77–1.43)	0.77	30 (3.1)	23 (2.3)	1.24 (0.74–2.05)	0.42
Cardiovascular death	47 (3.3)	49 (3.4)	0.96 (0.65–1.41)	0.82	10 (0.9)	11 (1.0)	0.93 (0.42–2.04)	0.86
Noncardiovascular death	27 (2.0)	24 (1.7)	1.20 (0.70–2.07)	0.50	20 (2.1)	12 (1.2)	1.52 (0.77–2.98)	0.23
Nonfatal myocardial infarction	21 (1.5)	28 (2.1)	0.92 (0.55–1.55)	0.76	19 (2.0)	13 (1.3)	1.73 (0.87–3.44)	0.12
Stroke	9 (0.6)	9 (0.6)	1.21 (0.50–2.91)	0.68	10 (0.9)	4 (0.4)	2.56 (0.80–8.15)	0.11
Major bleeding	21 (1.5)	26 (1.9)	0.77 (0.44–1.35)	0.37	13 (1.2)	14 (1.3)	0.93 (0.45–1.93)	0.85

Data are presented as n (%). DCB indicates drug‐coated balloon; DES, drug‐eluting stent; and HR, hazard ratio.

## DISCUSSION

The primary results of the present study suggest that the DCB strategy in PCI for ACS, based on real‐world data, achieved similar clinical outcomes to the DES strategy in the 1‐year postoperative period. Regardless of the localization of the culprit lesion or the ACS subtype, these outcomes were generally equivalent.

### DES Strategy in ACS

In the context of PCI for ACS, new‐generation DES has become the standard treatment. The clinical superiority of new‐generation DES over BMS and first‐generation DES has been reported, demonstrating advantages in target lesion revascularization and a reduced incidence of stent thrombosis.^15^, ^16^, ^17^ Based on these findings, the European guidelines in 2023 recommend new‐generation DES over BMS for both STEMI and non‐STEMI.^1^ In the prematched cohort of the present study, 94% of patients underwent the DES strategy; therefore, the DES strategy for ACS is widely accepted as the standard approach in real‐world clinical practice. However, the stent strategy in ACS is associated with factors that may lead to stent thrombosis, such as stent malapposition and delayed tissue coverage.^18^ In consideration of the adverse prognosis associated with stent‐related complications, there may be other optimal choices for PCI in ACS besides the stent strategy.

### DCB Strategy in ACS

Based on the differences in performance between BMS and DES and the presence of stent‐related complications, drug delivery to the vessel wall is crucial for the prevention of post‐PCI restenosis. In this context, the DCB strategy is considered to be rational because it has the potential to achieve this goal while overcoming stent‐related complications. In Japan, clinicians select the DCB strategy based on an expert consensus,^19^, ^20^ taking into account patient and lesion characteristics. Through the use of DCB, it is possible to deliver antiproliferative agents to the vessel wall without the need to place a permanent metal stent. This approach not only aids in restoring vascular function in the treated vessel and suppressing neointimal hyperplasia, but also has the potential to preserve the option for future coronary artery bypass grafting. These compelling characteristics provide ample rationale for examining the potential of the DCB strategy in ACS scenarios. In the prespecified analysis of the Basel Kosten Effektivitäts Trial–Drug‐Coated Balloons versus Drug‐eluting Stents in Small Vessel Interventions 2 (BASKET‐SMALL2) trial, the incidence of major adverse cardiac events, defined as cardiac death, nonfatal MI, and target vessel revascularization, did not significantly differ between the DCB and DES strategies in patients with ACS in the 1‐year follow‐up (6.3% versus 12.0%; *P*=0.088).^21^ The present study was a registry‐based investigation evaluating the 1‐year prognosis of the DCB strategy in PCI for ACS at 172 major facilities in Japan. Across all cohorts, the 1‐year incidence of all‐cause mortality consistently remained similar between the DCB and DES strategies. This suggests that the findings of high‐quality, small‐scale randomized controlled trials^10^, ^11^, ^21^ are applicable in real‐world clinical settings, and the present study has the potential to complement and extend these findings. Additionally, in this study, the DCB group exhibited a higher prevalence of cardiovascular risk factors, suggesting the presence of complex lesions or a high bleeding risk condition.^22^, ^23^ This finding suggests a potential tendency in clinical practice to preferentially use DCBs in cases with such clinical backgrounds, and its confirmation in a large‐scale ACS cohort represents a novel insight. In the present study, the DCB strategy was associated with a higher incidence of stroke and a lower incidence of bleeding events at 1 year in the overall cohort. These outcomes were not reproduced in other cohorts, warranting a cautious interpretation. These results may be attributed to the higher prevalence of cardiovascular comorbidities in the DCB group, resulting in a higher percentage of patients with more severe underlying conditions, as well as clinicians being more likely to opt for short‐duration dual antiplatelet therapy in cases treated with DCB alone. However, supporting evidence for this assumption is lacking, and further investigations are needed to confirm this hypothesis.

### Implementation of the DCB Strategy

Proper luminal patency before drug application is crucial in the DCB strategy. In the context of vascular reconstruction, general acceptance for the use of DCB is limited to cases in which successful predilation has been achieved, as outlined in consensus statements.^20^, ^24^ In the BASKET‐SMALL2 trial, which reported the long‐term efficacy and safety of DCB, ≈14% of participants were excluded from randomization due to flow‐limiting dissection or residual stenosis.^8^ The DCB strategy does not overcome the primary limitations of balloon angioplasty, including acute vessel recoil, dissection with flow limitation, and acute vessel closure. Additionally, if drug delivery to the vessel wall is hindered by evident thrombus, the DCB strategy may not be successful and, thus, the DES strategy is likely to be prioritized. The higher use of thrombus aspiration and distal protection devices in the DES group in this study indirectly suggests a preference for a DES strategy in clinical practice for lesions with a large thrombus burden. Furthermore, when using DCB for these lesions, it is essential to improve drug delivery to the vessel wall by removing as much thrombus as possible. Due to the nature of the registry, it was not possible to analyze the utilization rate of intravascular imaging in this study. However, in Japan, intravascular imaging is reimbursed, leading to high utilization rates. For example, in the Short and Optimal Duration of Dual Antiplatelet Therapy‐3 (STOPDAPT‐3) trial^25^ and Platelet rEactivity in patieNts with DrUg eLUting stent and balancing risk of bleeding and ischeMic event (PENDULUM) registry,^26^ both of which included patients with ACS undergoing PCI, the utilization rates of these devices were 93% and 94%, respectively, despite their use not being mandated in the study protocols. Similarly, other multicenter registry data in Japan^27^ have also consistently shown high utilization rates of intravascular imaging, with rates higher than 84%. In clinical practice, it is crucial to carefully evaluate these findings using the imaging modalities recommended in several consensus statements^28^, ^29^, ^30^ and to select each strategy cautiously. On the other hand, based on studies showing improved outcomes with delayed stent placement in primary PCI for STEMI, a new option has emerged: the staging use of DCB after the restoration of TIMI Flow Grade 3.^31^ Recent guidelines also report the use of DCB for non–ST‐segment–elevation myocardial infarction,^1^ contributing to growing anticipation for the DCB strategy.

### Limitations

There are a number of limitations that need to be addressed. The present study was conducted as registry‐based observational research. In this nonrandomized study, PS matching was used to adjust for selection bias; however, this method cannot fully address selection bias. Structural bias may still exist due to treatment switches occurring before patient identification. Furthermore, the lack of data on primary treatment intention in our database precludes an evaluation of the intention‐to‐treat effect of DCB relative to DES, highlighting the need for further investigations. During the matching process, nearly all DCB cases were successfully matched; therefore, the change in the hazard ratio depended on the outcomes of DES cases after matching. Additionally, in the primary analysis, within the matched cohort, the incidence of cardiogenic shock before the procedure was higher in the DES strategy than in the DCB strategy and the percentage of patients with STEMI was also higher in the former group. These biases may have affected the results obtained. Nevertheless, in the secondary analysis, which was matched by culprit lesions and ACS subtypes and in which covariates were well balanced, the results obtained were consistent across the 3 cohorts, except for non‐ST‐elevation ACS in the minor lesion cohort. The registry utilized also lacked information on procedural and lesion characteristics, such as thrombus grade and other lesion features, rendering it impossible to obtain details on lesion features that affected the selection of each treatment strategy. We conducted a stratified analysis based on the localization of culprit lesions; however, bias persisted in several factors, which may have led to an overestimation of the unfavorable prognosis associated with the DES strategy. In clinical practice, there are cases where, despite attempting the DCB strategy, a bailout stent implantation was implemented or cases where, based on imaging guidance, the DCB strategy was deemed unsuitable and, thus, the DES strategy was pursued. However, due to the characteristics of the registry, detailed data on these cases were lacking and, thus, they were not analyzed. It is reasonable to consider that the outcomes of previous randomized controlled trials and the present study were primarily applicable to a highly selected patient population, and it is essential to note that the present results may not be extrapolated to all ACS cases. Furthermore, it is important to emphasize that this registry did not include outcomes such as target lesion revascularization and target vessel revascularization, which are crucial for comparing the 2 strategies, thereby precluding any evaluation of these measures. Another limitation is that only a single type of paclitaxel‐coated balloon was available during the study period, while multiple DES products were on the market in Japan. Therefore, there was a potential bias in the treatment outcomes for ACS among the various DES used in this study. Moreover, the definition of bleeding events in the present study differed from the most commonly used definition proposed by the Bleeding Academic Research Consortium.^32^ One significant advantage of opting for the DCB strategy is the potential shortening of the duration of antiplatelet therapy. However, our registry lacked data on the duration of antiplatelet therapy, making it impossible to evaluate its potential impact on patient prognosis. In addition, our data were limited to 1 year and, thus, there were no long‐term outcomes. The present study also did not examine details related to the left ventricular ejection fraction, cardiac enzymes, medication at discharge, and the escalation of optimal medical therapy, which are considered to affect the long‐term prognosis of patients.

## CONCLUSIONS

During the 1‐year follow‐up, patients with ACS successfully treated with DCB achieved clinical outcomes similar to those undergoing treatment with DES. To confirm these results, a more comprehensive study with an extended follow‐up period and an assessment of individual lesion characteristics is necessary.

## Appendix

### J‐PCI (Japanese Percutaneous Coronary Intervention) Registry Investigators

Members of the Japanese Association of Cardiovascular Intervention and Therapeutics Scientific Committee: Shiro Uemura (Kawasaki Medical School), Yutaka Hikichi (Saga Medical Center Koseikan), Osamu Iida (Osaka Police Hospital), Yuji Ikari (Tokai University School of Medicine), Koichi Kaikita (University of Miyazaki), Yoshio Kobayashi (Chiba University Graduate School of Medicine), Toshiro Shinke (Showa University School of Medicine), Shinjo Sonoda (Saga University), Saeko Takahashi (Tokushukai Shonan Oiso Hospital), Kiyoshi Hibi (Yokohama City University Graduate School of Medicine), Yuichiro Maekawa (Hamamatsu University School of Medicine), and Shinichiro Yamada (Kitaharima Medical Center).

Members of the Japanese Association of Cardiovascular Intervention and Therapeutics Registry Committee: Tetsuya Amano (Aichi Medical University), Shun Kohsaka (Keio University School of Medicine), Osamu Iida (Osaka Police Hospital), Hideki Ishii (Gunma University Graduate School of Medicine), Kenichi Sakakura (Jichi Medical University), Toshiro Shinke (Showa University School of Medicine), Masato Nakamura (Toho University School of Medicine), and Tetsuya Matoba (Kyushu University).

Members of the Japanese Association of Cardiovascular Intervention and Therapeutics Working Subcommittee (J‐PCI): Shun Kohsaka (Keio University School of Medicine), Hideki Ishii (Gunma University Graduate School of Medicine), Kyohei Yamaji (Kyoto University Graduate School of Medicine), Hirohiko Ando (Aichi Medical University), Ayako Kunimura (Aichi Medical University), Kenichi Sakakura (Jichi Medical University), Takahiro Suzuki (St. Luke's International Hospital), Yuichiro Mori (Kyoto University Graduate School of Medicine), and Wada Hideki (Juntendo University Shizuoka Hospital).

Members of the Japanese Association of Cardiovascular Intervention and Therapeutics Working Subcommittee (J‐PCIOUTCOME): Shun Kohsaka (Keio University School of Medicine), Hideki Ishii (Gunma University Graduate School of Medicine), Kazuyuki Ozaki (Niigata University Graduate School of Medical and Dental Sciences), Yuetsu Kikuta (Fukuyama Cardiovascular Hospital), Kunii Hiroyuki (Ohara General Hospital), Syoichi Kuramitsu (Sapporo Cardiovascular Clinic), Mitsuaki Sawano (Yale/YNHH Center for Outcomes Research and Evaluation), Jun Takahashi (Tohoku University Graduate School of Medicine), Toshiharu Takeuchi (Asahikawa Medical University), Yohei Numasawa (Keio University School of Medicine), Tetsuya Matoba (Kyushu University), Kyohei Yamaji (Kyoto University Graduate School of Medicine), and Tetsu Watanabe (Yamagata University School of Medicine).

## Sources of Funding

Agency for Medical Research and Development, Grant/Award Number: 17ek0210097h0001; Japan Society for the Promotion of Science, Grant (KAKENHI)/Award Number: 21K08064.

## Disclosures

Dr Kyohei Yamaji received investigator‐initiated grant funding from Abbott. Dr Shun Kohsaka received investigator‐initiated grant funding from Bayer and Daiichi Sankyo and personal fees from Bayer and Bristol‐Myers Squibb. Dr Hideki Ishii received lecture fees from AstraZeneca, Bayer, Bristol‐Myers Squibb, Daiichi‐Sankyo, Kowa, MSD, Mitsubishi Tanabe, Mochida, and Otsuka Pharma. Dr Tetsuya Amano received lecture fees from Astellas Pharma, AstraZeneca, Bayer, Daiichi Sankyo, and Bristol‐Myers Squibb. Dr Ken Kozuma received lecture fees from Boston Scientific, Abbott Medical, Medtronic, Otsuka, Takeda, Daiichi‐Sankyo, Amgen, Novartis, Behringer, Bayer, Life Science Institute, Mochida, and Zeon Medical, and scholarship funds from Boston Scientific and Abbott Medical. The remaining authors declare no conflicts of interest.

## Supporting information

Data S1
Tables S1–S9
Figures S1–S2
