# Supplementary material for: Successful or Uncomplicated Use of Drug‐Coated Balloon Versus Drug‐Eluting Stent Strategies for De Novo Culprit Lesions in Acute Coronary Syndromes: Insights from a Nationwide Registry in Japan
Source: J Am Heart Assoc. 2025 May 29;14(11):e038071. doi: 10.1161/JAHA.124.038071 (PMC12229198; doi:10.1161/JAHA.124.038071)
Supplement: Supplementary file 1 — Data S1 Tables S1–S9 Figures S1–S2 [file JAH3-14-e038071-s001.pdf]

SUPPLEMENTAL MATERIAL

Data S1. Supplemental Methods.

Overview and Details of the J-PCI OUTCOME Registry

The J-PCI OUTCOME study is derived from the J-PCI registry, a prospective, multicenter nationwide PCI registry led by the Japanese Association of Cardiovascular Intervention and Therapeutics (CVIT). The J-PCI registry aims to provide national, regional, and institutional data on general patient characteristics, including background, clinical findings, angiographic and procedural details, and in-hospital outcomes, serving as a foundation for further quality improvement. Since 2013, the registry has been incorporated into the National Clinical Data System, a nationwide prospective web-based registry linked to medical and surgical board certification programs. Public and private hospitals are both eligible to register data in this registry, and it is estimated that approximately 90% of PCI procedures performed in Japan are captured in the J-PCI registry, a significantly higher percentage than annual insurance claims data.

In December 2017, more than 1,000 representative PCI centers from all 47 prefectures in Japan were invited to participate in the J-PCI OUTCOME study. Eligibility criteria required each standardized operational facility to perform more than 200 PCI procedures annually. A total of 599 hospitals responded, and 172 institutions that registered more than 10 patients during the study period were included in the final analysis to ensure data quality.

A previous study[12] reported comparisons between the J-PCI OUTCOME cohort (172 participating facilities) and the J-PCI cohort (representing over 90% of PCI centers in Japan) in terms of patient backgrounds and PCI characteristics. These analyses showed that the percentage of patients with ACS, chronic kidney disease (CKD), or proximal coronary lesions was higher in the J-PCI OUTCOME cohort, while the percentage of patients with prior PCI or myocardial infarction was lower than in the J-PCI cohort. No significant differences were observed for other parameters.

Table S1. Covariate Definitions.

Covariate	Definition
Age	Patient's age in years at the time of the index percutaneous coronary intervention
Male	Patients with male sex identified at birth
History of PCI	Patients with a history of one or more prior percutaneous coronary interventions
History of CABG	Patients with a history of one or more previous coronary artery bypass grafting procedures
Prior myocardial infarction	Patients previously diagnosed with myocardial infarction
Prior heart failure	Patients previously diagnosed with heart failure. Cases of left ventricular dysfunction without accompanying symptoms were excluded
Hypertension	At least one of the following criteria is met based on the Japanese Society of Hypertension 2009 guidelines:<br>(a) Systolic blood pressure ≥140 mmHg<br>(b) Diastolic blood pressure ≥90 mmHg<br>(c) Undergoing treatment with antihypertensive agents
Diabetes	At least one of the following criteria is met:<br>(a) Fasting blood glucose ≥126 mg/dL<br>(b) Random blood glucose ≥200 mg/dL<br>(c) HbA1c ≥6.5 (as per the Japanese formula)<br>(d) 2-h 75 g OGTT blood glucose ≥200 mg/dL<br>(e) Treatment with oral antidiabetic agents, insulin, or incretin medication
Dyslipidemia	Any of the following is met based on the Japan Atherosclerosis Society (JAS) Guidelines for the Prevention of Atherosclerotic Cardiovascular Diseases 2012<br>(a) LDL cholesterol ≥140 mg/dL<br>(b) HDL cholesterol <40 mg/dL<br>(c) Triglycerides ≥150 mg/dL<br>(d) LDL cholesterol is calculated using the Friedewald formula (TC–HDL-C–TG/5) (when TG <400 mg/dL). When TG is ≥400 mg/dL or using postprandial blood, non-HDL-C (TC–HDL-C) needs to be used
Smoking	All patients with a history of smoking within the previous year
Chronic kidney disease	At least one of the following criteria needs to be met (Japanese Society of Nephrology CKD Treatment Guidelines 2009):<br>(a) Proteinuria<br>(b) Serum creatinine ≥1.3 mg/dL<br>(c) eGFR ≤60 ml/min/1.73 m^2<br>(e) GFR = 194 × age − 0.23 × Cre − 0.1154 [women × 0.742])
Dialysis	Patients undergoing hemodialysis or peritoneal dialysis

Chronic obstructive lung disease	Patients with a forced expiratory volume in one second (FEV1) of 70% or less or those receiving inhaled steroids or bronchodilators (inhaled or oral), with the exclusion of patients with bronchial asthma
Peripheral artery disease	Patients with a history of non-cardiac vascular lesions, including aortic aneurysm or peripheral vessel stenosis (such as renal, mesenteric, iliac, and femoral arteries) with more than 50% narrowing, including:<br>(a) Symptoms of intermittent claudication<br>(b) Ankle-brachial index (ABI) ≤0.9<br>(c) History of claudication, amputation, bypass surgery, or vascular reconstruction due to arterial stenosis or occlusion
Baseline hemoglobin	Pre-procedural hemoglobin level at the date and time closest to PCI
ST-elevation myocardial infarction	Patients with persistent myocardial ischemia symptoms accompanied by elevated cardiac biomarkers and elevated ST segments on ECG: refer to elevated creatine kinase (CK) or CK-MB levels [two-fold higher than normal values] or elevated troponin levels [≥99th percentile] with T elevation on two or more contiguous leads (≥0.2 mV in a precordial lead at the J point or ≥0.1 mV in a limb lead), a new left bundle branch block, or posterior myocardial infarction on a 12-lead ECG.
Non-ST-elevation myocardial infarction	Patients with persistent myocardial ischemia symptoms accompanied by elevated cardiac biomarkers without elevated ST segments on a 12-lead ECG.
Unstable angina	At least one of the following is met:<br>(a) New-onset angina: angina that manifested within the past month<br>(b) Increasing angina: angina that worsened within the past month<br>(c) Resting angina: persistent angina at rest or angina that markedly restricts daily life (symptoms triggered by walking tens of meters or one flight of stairs)<br>(d) Post-infarction angina: persistent angina within one month following a myocardial infarction event with/without the involvement of elevated ST segments on ECG or cardiac biomarkers, based upon which angina is defined as STEMI or NSTEMI, respectively
Non-ST-elevation acute coronary syndrome	Among acute coronary syndrome, conditions excluding ST-elevation myocardial infarction.
Cardiopulmonary arrest within 24 h	Patients with cardiac arrest (presenting with asystole, ventricular fibrillation, or pulseless ventricular tachycardia) requiring cardiopulmonary resuscitation within 24 hours before undergoing percutaneous coronary intervention
Cardiogenic shock within 24 h	Patients clinically diagnosed with circulatory failure within 24 hours prior to percutaneous coronary intervention (PCI), meeting one of the following criteria:<br>(a) Despite maximal therapy, systolic blood pressure <80 mmHg and/or a cardiac index <1.8 l/min/m^2.<br>(b) Intravenous administration of inotropes or IABP support is required to maintain systolic blood pressure >80 mmHg and a cardiac index >1.8 l/min/m^2
Acute heart failure within 24 h	Patients diagnosed with a state of acute heart failure, experiencing symptoms even at rest or with mild exertion (NYHA class IV equivalent) within 24 hours prior to the percutaneous coronary intervention

Number of diseased vessels	The number of major native coronary vessel systems with significant stenotic lesions, defined as having an AHA classification of 75% or more.
Left main disease	Left main disease is present when stenosis affects 50% or more of the vessel diameter

AHA, American Heart Association; CABG, coronary artery bypass graft; CKD, chronic kidney disease; eGFR, estimated glomerular filtration rate; HbA1c, hemoglobin A1c; HDL, high-density lipoprotein; IABP, intra-aortic balloon pumping; LDL, low-density lipoprotein; NSTEMI, non-ST-elevation myocardial infarction; NYHA, New York Heart Association; OGTT, oral glucose tolerance test; PCI, percutaneous coronary intervention; STEMI, ST-elevation myocardial infarction; TC, total cholesterol; TG, triglyceride.

Table S2. Standardized Differences in Baseline Characteristics Before and After Propensity Score Matching.

	Non-matching	Matching
**Clinical characteristics**		
Age, years	0.02	0.00
Male sex, n (%)	-0.07	0.00
History of PCI, n (%)	-0.32	-0.01
History of CABG, n (%)	-0.11	-0.04
Prior myocardial infarction, n (%)	-0.32	-0.01
Prior heart failure, n (%)	-0.09	-0.01
Hypertension, n (%)	-0.16	0.03
Diabetes, n (%)	-0.10	0.01
Dyslipidemia, n (%)	-0.13	0.02
Smoking, n (%)	0.05	-0.01
Chronic kidney disease, n (%)	-0.08	0.01
Dialysis, n (%)	-0.13	0.00
Chronic obstructive lung disease, n (%)	0.02	-0.03
Peripheral artery disease, n (%)	-0.05	0.02
Baseline hemoglobin (g/dL)	0.10	0.03
**Clinical presentations, n (%)**		
ST-elevation myocardial infarction	0.23	0.22
Cardiopulmonary arrest within 24 h	0.04	0.01
Cardiogenic shock within 24 h	0.10	0.1
Acute heart failure within 24 h	0.07	0.09
Number of diseased vessels		

1	-0.10	-0.04
2	0.00	0.03
3	-0.03	0.02
Left main disease	0.04	0.06
**Preprocedural medications, n (%)**		
Antiplatelets	-0.10	0.08
Aspirin	0.03	0.07
Clopidogrel	-0.12	-0.03
Prasugrel	0.14	0.1
Ticagrelor	0.01	0.02
Oral anticoagulants	-0.02	-0.01
Warfarin	-0.02	0.00
Direct oral anticoagulant	0.00	-0.01
**Preprocedural mechanical circulatory assist device, n (%)**		
ECMO	0.04	0.03
Impella	0.04	0.04
IABP	0.05	0.06
**Lesion locations, n (%)**		
Right coronary artery		
Segment 1	0.16	0.18
Segment 2	0.13	0.17
Segment 3	0.08	0.14
Segment 4	-0.25	-0.2
Left main trunk	0.08	0.11
Left anterior descending artery		

Segment 6	0.17	0.15
Segment 7	0.16	0.18
Segment 8	-0.11	-0.09
Segment 9	-0.51	-0.51
Segment 10	-0.15	-0.17
Left circumflex artery		
Segment 11	-0.09	-0.08
Segment 12 or HL	-0.22	-0.23
Segment 13	-0.05	-0.03
Segment 14	-0.15	-0.15
Segment 15	-0.10	-0.11
**Devices used during PCI, n (%)**		
Rotational atherectomy	-0.13	-0.10
Thrombus aspiration	0.25	0.17
Distal protection device	0.19	0.17

CABG, Coronary Artery Bypass Graft; DCB, Drug-coated Balloon; DES, Drug-eluting Stent; eGFR, Estimated Glomerular Filtration Rate; ECMO, Extracorporeal Membrane Oxygenation; HL, High Lateral Branch; IABP, Intra-aortic Balloon Pumping; PCI, Percutaneous Coronary Intervention.

Table S3. Baseline Characteristics Before and After Propensity Score Matching in the Major Lesion Cohort.

	Non-matching				Matching		
	All	DES	DCB	Std. diff	DES	DCB	Std. diff
	(n=38,781)	(n=37,133)	(n=1,648)		(n=1,537)	(n=1,537)	
**Clinical characteristics**							
Age, years	69 ± 13	69 ± 13	69 ± 13	0.03	69 ± 13	69 ± 13	-0.01
Male sex, n (%)	29,515 (76.1)	28,281 (76.2)	1,234 (74.9)	0.02	1,165 (75.8)	1,152 (75.0)	0.02
History of PCI, n (%)	4,867 (12.6)	4,402 (11.9)	465 (28.3)	-0.36	441 (28.7)	441 (28.7)	0.00
History of CABG, n (%)	516 (1.3)	463 (1.3)	53 (3.2)	-0.12	32 (2.1)	50 (3.3)	-0.06
Prior myocardial infarction, n (%)	3,345 (8.7)	3,088 (8.4)	257 (15.7)	-0.19	229 (14.9)	245 (15.9)	-0.02
Prior heart failure, n (%)	2,423 (6.3)	2,269 (6.2)	154 (9.4)	-0.1	143 (9.3)	151 (9.8)	-0.01
Hypertension, n (%)	26,766 (73.0)	25,571 (72.8)	1,195 (76.4)	-0.07	1,181 (76.8)	1,174 (76.4)	0.01
Diabetes, n (%)	13,464 (36.7)	12,809 (36.5)	655 (41.9)	-0.09	649 (42.2)	642 (41.8)	0.01
Dyslipidemia, n (%)	23,306 (63.5)	22,270 (63.4)	1,036 (66.2)	-0.05	1,033 (67.2)	1,023 (66.6)	0.01
Smoking, n (%)	15,017 (40.9)	14,453 (41.2)	564 (36.0)	0.09	548 (35.7)	549 (35.7)	0.00
Chronic kidney disease, n (%)	6,607 (18.0)	6,248 (17.8)	359 (22.9)	-0.11	336 (21.9)	344 (22.4)	-0.01
Dialysis, n (%)	1,205 (3.3)	1,090 (3.1)	115 (7.4)	-0.17	104 (6.8)	111 (7.2)	-0.01
Chronic obstructive lung disease, n (%)	965 (2.6)	931 (2.7)	34 (2.2)	0.03	24 (1.6)	34 (2.2)	-0.04
Peripheral artery disease, n (%)	1,421 (3.9)	1,337 (3.8)	84 (5.4)	-0.06	92 (6.0)	83 (5.4)	0.02
Baseline hemoglobin (g/dL)	13.7 ± 2.1	13.8 ± 2.1	13.3 ± 2.3	0.16	13.7 ± 2.22	13.4 ± 2.29	0.09
**Clinical presentations, n (%)**							
ST-elevation myocardial infarction	21,736 (56.0)	21,060 (56.7)	676 (41.0)	0.26	767 (49.9)	627 (40.8)	0.15
Cardiopulmonary arrest within 24 h	1,567 (4.1)	1,506 (4.1)	61 (3.7)	0.01	45 (2.9)	56 (3.7)	-0.03
Cardiogenic shock within 24 h	2,685 (7.0)	2,603 (7.0)	82 (5.0)	0.07	86 (5.6)	77 (5.0)	0.02
Acute heart failure within 24 h	2,793 (7.2)	2,693 (7.3)	100 (6.1)	0.04	101 (6.6)	90 (5.9)	0.02
Number of diseased vessels							
1	26,356 (68.0)	25,232 (68.0)	1,124 (68.2)	0.00	1023 (66.6)	1039 (67.6)	-0.02
2	8,411 (21.7)	8,077 (21.8)	334 (20.3)	0.03	344 (22.4)	316 (20.6)	0.04
3	4,014 (10.4)	3,824 (10.3)	190 (11.5)	-0.03	170 (11.1)	182 (11.8)	-0.02
Left main disease	902 (2.3)	868 (2.3)	34 (2.1)	0.02	42 (2.7)	32 (2.1)	0.03
**Preprocedural medications, n (%)**							
Antiplatelets	33,525 (86.4)	32,130 (86.5)	1,395 (84.6)	0.04	1,375 (89.5)	1,304 (84.8)	0.12

Aspirin	32,749 (97.5)	31,401 (84.6)	1,348 (81.8)	0.06	1,338 (87.1)	1,259 (81.9)	0.12
Clopidogrel	5,619 (16.7)	5,273 (14.2)	346 (21.0)	-0.15	279 (18.2)	324 (21.1)	-0.06
Prasugrel	25,383 (75.6)	24,460 (65.9)	923 (56.0)	0.17	967 (62.9)	868 (56.5)	0.11
Ticagrelor	40 (0.1)	38 (0.1)	2 (0.1)	0.00	1 (0.1)	2 (0.1)	-0.02
Oral anticoagulants	1,622 (4.2)	1,543 (4.2)	79 (4.8)	-0.03	73 (4.7)	73 (4.7)	0.00
Warfarin	583 (34.5)	554 (1.5)	29 (1.8)	-0.02	33 (2.1)	27 (1.8)	0.02
Direct oral anticoagulant	1,125 (2.9)	1,073 (2.9)	52 (3.2)	-0.01	42 (2.7)	48 (3.1)	-0.02
**Preprocedural mechanical circulatory assist device, n (%)**							
ECMO	196 (0.5)	192 (0.5)	4 (0.2)	0.03	6 (0.4)	4 (0.3)	0.02
Impella	58 (0.2)	58 (0.2)	0 (0%)	0.04	3 (0.2)	0 (0)	0.04
IABP	593 (1.5)	572 (1.5)	21 (1.3)	0.02	29 (1.9)	20 (1.3)	0.04
**Lesion locations, n (%)**							
Right coronary artery							
Segment 1	4,970 (12.8)	4,817 (13.0)	153 (9.3)	0.09	212 (13.8)	144 (9.4)	0.11
Segment 2	5,078 (13.1)	4,899 (13.2)	179 (10.9)	0.06	200 (13.0)	162 (10.5)	0.06
Segment 3	3,848 (9.92)	3,688 (9.9)	160 (9.7)	0.01	175 (11.4)	151 (9.8)	0.04
Left main trunk	838 (2.2)	819 (2.2)	19 (1.2)	0.06	42 (2.7)	17 (1.1)	0.09
Left anterior descending artery							
Segment 6	10,704 (27.6)	10,285 (27.7)	419 (25.4)	0.04	390 (25.4)	388 (25.2)	0.00
Segment 7	8,437 (21.8)	8,116 (21.9)	321 (19.5)	0.05	335 (21.8)	301 (19.6)	0.04
Left circumflex artery							
Segment 11	1,926 (5.0)	1,746 (4.7)	180 (10.9)	-0.20	76 (4.9)	168 (10.9)	-0.19
Segment 13	2,980 (7.7)	2,763 (7.4)	217 (13.2)	-0.16	107 (7.0)	206 (13.4)	-0.18
**Devices used during PCI, n (%)**							
Rotational atherectomy	547 (1.4)	463 (1.3)	84 (5.1)	-0.2	23 (1.5)	80 (5.2)	-0.19
Thrombus aspiration	14,115 (36.4)	13,691 (36.9)	424 (24.7)	0.19	495 (32.2)	397 (25.8)	0.11
Distal protection device	2,751 (7.1)	2,709 (7.3)	42 (2.6)	0.17	93 (6.1)	40 (2.6)	0.13

Data are presented as n (%) or means (standard deviations).

CABG, Coronary Artery Bypass Graft; DCB, Drug-coated Balloon; DES, Drug-eluting Stent; eGFR, Estimated Glomerular Filtration Rate; ECMO, Extracorporeal Membrane Oxygenation; HL, High Lateral Branch; IABP, Intra-aortic Balloon Pumping; PCI, Percutaneous Coronary Intervention; Std. diff, Standardized Difference.

Table S4. Baseline Characteristics Before and After Propensity Score Matching in the Minor Lesion Cohort.

	Non-matching				Matching		
	All	DES	DCB	Std. diff	DES	DCB	Std. diff
	(n=4,608)	(n=3,489)	(n=1,119)		(n=1,069)	(n=1,069)	
**Clinical characteristics**							
Age, years	69 ± 12	69 ± 13	69 ± 12	-0.01	70 ± 12	69 ± 12	0.03
Male sex, n (%)	3,611 (78.4)	2,764 (79.2)	847 (75.7)	0.07	788 (73.7)	815 (76.2)	-0.05
History of PCI, n (%)	885 (19.2)	635 (18.3)	250 (22.3)	-0.09	217 (20.3)	241 (22.5)	-0.04
History of CABG, n (%)	95 (2.1)	63 (1.8)	32 (2.9)	-0.06	17 (1.6)	32 (3.0)	-0.08
Prior myocardial infarction, n (%)	551 (12.0)	391 (11.3)	160 (14.4)	-0.08	134 (12.5)	157 (14.7)	-0.05
Prior heart failure, n (%)	292 (6.4)	208 (6.0)	84 (7.5)	-0.05	60 (5.6)	80 (7.5)	-0.06
Hypertension, n (%)	3,462 (78.5)	2,602 (78.1)	860 (79.9)	-0.04	878 (82.1)	854 (79.9)	0.05
Diabetes, n (%)	1,669 (37.9)	1,265 (38.0)	404 (37.5)	0.00	401 (37.5)	402 (37.6)	0.00
Dyslipidemia, n (%)	2,940 (66.7)	2,196 (65.9)	744 (69.1)	-0.06	757 (70.8)	744 (69.6)	0.02
Smoking, n (%)	1,675 (38.0)	1,316 (39.5)	359 (33.4)	0.1.0	340 (31.8)	356 (33.3)	-0.03
Chronic kidney disease, n (%)	754 (17.1)	578 (17.3)	176 (16.4)	0.02	173 (16.2)	174 (16.3)	0.00
Dialysis, n (%)	144 (3.3)	103 (3.1)	41 (3.8)	-0.03	31 (2.9)	40 (3.7)	-0.04
Chronic obstructive lung disease, n (%)	108 (2.5)	85 (2.6)	23 (2.1)	0.02	16 (1.5)	22 (2.1)	-0.04
Peripheral artery disease, n (%)	186 (4.2)	145 (4.4)	41 (3.8)	0.02	39 (3.7)	41 (3.84)	-0.01
Baseline hemoglobin (g/dL)	13.9 ± 2.1	13.9 ± 2.1	13.8 ± 2.1	0.03	13.9 ± 2.04	13.8 ± 2.13	0.03
**Clinical presentations, n (%)**							
ST-elevation myocardial infarction	1,807 (39.2%)	1,441 (41.3%)	366 (32.7%)	0.14	452 (42.3)	352 (32.9)	0.16
Cardiopulmonary arrest within 24 h	70 (1.5)	56 (1.6)	14 (1.3)	0.02	9 (0.8)	13 (1.2)	-0.03
Cardiogenic shock within 24 h	89 (1.9)	76 (2.2)	13 (1.2)	0.06	19 (1.8)	13 (1.2)	0.04
Acute heart failure within 24 h	125 (2.7)	103 (3.0)	22 (2.0)	0.05	36 (3.38)	21 (1.97)	0.07
Number of diseased vessels							
1	3,074 (66.7)	2,294 (65.7)	780 (69.7)	-0.07	708 (66.2)	748 (70.0)	-0.07
2	1,078 (23.4)	841 (24.1)	237 (21.2)	0.06	258 (24.1)	223 (20.9)	0.06
3	456 (9.9)	354 (10.1)	102 (9.1)	-0.39	103 (9.6)	98 (9.2)	0.01
Left main disease	27 (0.6)	21 (0.6)	6 (0.5)	-0.08	3 (0.3)	6 (0.6)	-0.04
**Preprocedural medications, n (%)**							
Antiplatelets	4,051 (87.9)	3,069 (88.0)	982 (87.8)	0.01	956 (89.4)	942 (88.1)	0.03

Aspirin	3,962 (97.7)	3,003 (86.1)	959 (85.7)	0.01	935 (87.5)	923 (86.3)	0.03
Clopidogrel	756 (18.6)	556 (15.9)	200 (17.9)	-0.04	170 (15.9)	188 (17.6)	-0.04
Prasugrel	2,928 (72.2)	2,255 (64.6)	673 (60.1)	0.08	689 (64.5)	650 (60.8)	0.06
Ticagrelor	1 (0.1)	1 (0.1)	0 (0%)	0.02	1 (0.1)	0 (0)	0.03
Oral anticoagulants	202 (4.38%)	150 (4.3%)	52 (4.6%)	-0.01	52 (4.9)	51 (4.7)	0.00
Warfarin	84 (41.0%)	62 (1.8%)	22 (2.0%)	-0.01	18 (1.7)	22 (2.1)	-0.02
Direct oral anticoagulant	123 (2.7)	93 (2.7)	30 (2.7)	0.00	29 (2.7)	24 (2.2)	0.02
**Preprocedural mechanical circulatory assist device, n (%)**							
ECMO	4 (0.1)	4 (0.1)	0 (0)	0.03	1 (0.1)	0 (0)	0.03
Impella	1 (0.1)	1 (0.1)	0 (0)	0.02	1 (0.1)	0 (0)	0.03
IABP	14 (0.3)	14 (0.4)	0 (0)	0.06	6 (0.6)	0 (0)	0.08
**Lesion locations, n (%)**							
Right coronary artery							
Segment 4	1,087 (23.6)	871 (25.0)	216 (19.3)	0.11	286 (26.8)	208 (19.4)	0.14
Left anterior descending artery							
Segment 8	253 (5.5)	205 (5.9)	48 (4.3)	0.06	65 (6.1)	47 (4.4)	0.06
Segment 9	1,158 (25.1)	703 (20.1)	455 (40.7)	-0.39	197 (18.4)	428 (40.0)	-0.42
Segment 10	94 (2.0)	51 (1.5)	43 (3.8)	-0.13	15 (1.4)	43 (4.02)	-0.14
Left circumflex artery							
Segment 12 or HL	1,085 (23.5)	885 (25.4)	200 (17.9)	0.15	258 (24.1)	189 (17.7)	0.13
Segment 14	582 (12.6)	480 (13.8)	102 (9.1)	0.12	159 (14.9)	100 (9.35)	0.13
Segment 15	349 (7.6)	294 (8.4)	55 (4.9)	0.11	89 (8.3)	54 (5.1)	0.10
**Devices used during PCI, n (%)**							
Rotational atherectomy	30 (0.07)	21 (0.6)	9 (0.8)	-0.02	7 (0.7)	8 (0.7)	-0.01
Thrombus aspiration	1,106 (24.0)	922 (26.4)	184 (16.4)	0.19	268 (25.1)	181 (16.9)	0.16
Distal protection device	29 (0.6)	27 (0.8)	2 (0.2)	0.06	6 (0.6)	2 (0.2)	0.05

Data are presented as n (%) or means (standard deviations).

CABG, Coronary Artery Bypass Graft; DCB, Drug-coated Balloon; DES, Drug-eluting Stent; eGFR, Estimated Glomerular Filtration Rate; ECMO, Extracorporeal Membrane Oxygenation; HL, High Lateral Branch; IABP, Intra-Aortic Balloon Pumping; PCI, Percutaneous Coronary Intervention; Std. diff, Standardized Difference.

Table S5. Baseline Characteristics Before and After Propensity Score Matching in STEMI in the Major Lesion Cohort.

	Non-matching				Matching		
	All	DES	DCB	Std. diff	DES	DCB	Std. diff
	(n=21,736)	(n=21,060)	(n=676)		(n=627)	(n=627)	
**Clinical characteristics**							
Age, years	69 ± 13	69 ± 13	67 ± 13	0.09	67 ±14	67 ± 13	-0.03
Male sex, n (%)	16,740 (77.0)	16,208 (77.0)	532 (78.7)	-0.03	490 (78.1)	492 (78.5)	-0.01
History of PCI, n (%)	1,489 (6.9)	1,381 (6.6)	108 (16.1)	-0.26	107 (17.1)	104 (16.6)	0.01
History of CABG, n (%)	113 (0.5)	104 (0.5)	9 (1.3)	-0.08	6 (1.0)	9 (1.4)	-0.04
Prior myocardial infarction, n (%)	1,247 (5.8)	1,175 (5.6)	72 (10.7)	-0.16	74 (11.8)	71 (11.3)	0.01
Prior heart failure, n (%)	772 (3.6)	733 (3.5)	39 (5.8)	-0.09	29 (4.6)	39 (6.2)	-0.06
Hypertension, n (%)	14,270 (69.9)	13,811 (69.8)	459 (72.3)	-0.04	461 (73.5)	454 (72.4)	0.02
Diabetes, n (%)	7,088 (34.7)	6,837 (34.6)	251 (39.5)	-0.08	248 (39.6)	246 (39.2)	0.01
Dyslipidemia, n (%)	12,434 (60.9)	12,017 (60.8)	417 (65.7)	-0.08	429 (68.4)	411 (65.6)	0.05
Smoking, n (%)	9,105 (44.6)	8,845 (44.7)	260 (40.9)	0.06	252 (40.2)	256 (40.8)	-0.01
Chronic kidney disease, n (%)	3,340 (16.4)	3,211 (16.2)	129 (20.3)	-0.08	115 (18.3)	127 (20.3)	-0.04
Dialysis, n (%)	347 (1.70)	315 (1.59)	32 (5.04)	-0.17	26 (4.2)	32 (5.1)	-0.04
Chronic obstructive lung disease, n (%)	493 (2.4)	484 (2.5)	9 (1.4)	0.06	11 (1.8)	9 (1.4)	0.02
Peripheral artery disease, n (%)	580 (2.8)	558 (2.8)	22 (3.5)	-0.03	25 (4.0)	22 (3.5)	0.02
Baseline hemoglobin (g/dL)	14.0 ± 2.1	14.0 ± 2.1	13.7 ± 2.3	0.09	14.0 ± 2.1	13.7 ± 2.3	0.09
**Clinical presentations, n (%)**							
Cardiopulmonary arrest within 24 h	1,251 (5.9)	1,208 (5.8)	43 (6.4)	-0.02	27 (4.3)	40 (6.4)	-0.08
Cardiogenic shock within 24 h	2,224 (10.3)	2,169 (10.3)	55 (8.2)	0.06	48 (7.7)	52 (8.3)	-0.02
Acute heart failure within 24 h	1,989 (9.2)	1,934 (9.2)	55 (8.2)	0.03	56 (8.9)	50 (8.0)	0.03
Number of diseased vessels							
1	14,906 (68.6)	14,440 (68.6)	466 (68.9)	-0.01	429 (68.4)	428 (68.3)	0.00
2	4,645 (21.4)	4,511 (21.4)	134 (19.8)	0.03	144 (23.0)	127 (20.3)	0.05
3	2,185 (10.1)	2,109 (10.0)	76 (11.2)	-0.03	54 (8.6)	72 (11.5)	-0.08
Left main disease	413 (1.9)	397 (1.9)	16 (2.4)	-0.03	13 (2.1)	15 (2.4)	-0.02
**Preprocedural medications, n (%)**							
Antiplatelets	18,278 (84.1)	17,745 (84.3)	533 (78.8)	0.12	544 (86.8)	495 (78.9)	0.18
Aspirin	17,964 (82.6)	17,439 (82.8)	525 (77.7)	0.11	533 (85.0)	488 (77.8)	0.16
Clopidogrel	1,812 (8.3)	1,747 (8.3)	65 (9.6)	-0.04	53 (8.5)	56 (8.9)	-0.01

Prasugrel	15,334 (70.5)	14,904 (70.8)	430 (63.6)	0.13	453 (72.2)	405 (64.6)	0.14
Ticagrelor	15 (0.1)	14 (0.1)	1 (0.1)	-0.02	0 (0)	1 (0.2)	-0.06
Oral anticoagulants	614 (2.8)	595 (2.8)	19 (2.8)	0.00	9 (1.4)	17 (2.7)	-0.08
Warfarin	226 (1.0)	217 (1.0)	9 (1.3)	-0.02	4 (0.6)	9 (1.4)	-0.07
Direct oral anticoagulant	434 (2.0)	422 (2.0)	12 (1.8)	0.01	5 (0.8)	10 (1.6)	-0.06
**Preprocedural mechanical circulatory assist device, n (%)**							
ECMO	163 (0.7)	161 (0.8)	2 (0.3)	0.05	3 (0.5)	2 (0.3)	0.02
Impella	46 (0.2)	46 (0.2)	0 (0)	0.05	1 (0.2)	0 (0)	0.04
IABP	429 (2.0)	415 (2.0)	14 (2.1)	-0.01	13 (2.1)	14 (2.2)	-0.01
**Lesion locations, n (%)**							
Right coronary artery							
Segment 1	3,339 (15.4)	3,255 (15.5)	84 (12.4)	0.07	97 (15.5)	77 (12.3)	0.07
Segment 2	3,146 (14.5)	3,050 (14.5)	96 (14.2)	0.01	84 (13.4)	86 (13.7)	-0.01
Segment 3	2,423 (11.1)	2,340 (11.1)	83 (12.3)	-0.03	73 (11.6)	77 (12.3)	-0.02
Left main trunk	316 (1.5)	313 (1.5)	3 (0.4)	0.08	8 (1.3)	3 (0.5)	0.07
Left anterior descending artery							
Segment 6	6,170 (28.4)	5,992 (28.5)	178 (26.3)	0.04	171 (27.3)	167 (26.6)	0.01
Segment 7	4,599 (21.2)	4,464 (21.2)	135 (20.0)	0.02	149 (23.8)	129 (20.6)	0.06
Left circumflex artery							
Segment 11	610 (2.8)	583 (2.8)	27 (4.0)	-0.06	12 (1.9)	21 (3.4)	-0.08
Segment 13	1,133 (5.2)	1,063 (5.1)	70 (10.4)	-0.17	33 (5.3)	67 (10.7)	-0.17
**Devices used during PCI, n (%)**							
Rotational atherectomy	84 (0.4)	73 (0.3)	11 (1.6)	-0.12	1 (0.2)	9 (1.4)	-0.14
Thrombus aspiration	12,041 (55.4)	11,703 (55.6)	338 (50.0)	0.09	357 (56.9)	318 (50.7)	0.1
Distal protection device	1,939 (8.9)	1,912 (9.1)	27 (4.0)	0.16	54 (8.6)	25 (4.0)	0.15

Data are presented as n (%) or means (standard deviations).

CABG, coronary artery bypass graft; DCB, drug-coated balloon; DES, drug-eluting stent; eGFR, estimated glomerular filtration rate; ECMO, extracorporeal membrane oxygenation; IABP, intra-aortic balloon pumping; PCI, percutaneous coronary intervention; Std. diff, standardized difference.

Table S6. Baseline Characteristics Before and After Propensity Score Matching in STEMI in the Minor Lesion Cohort.

	Non-matching				Matching		
	All	DES	DCB	Std. diff	DES	DCB	Std. diff
	(n=1,807)	(n=1,441)	(n=366)		(n=352)	(n=352)	
**Clinical characteristics**							
Age, years	68 ± 13	68 ± 13	67 ± 13	0.03	67 ± 13	67 ± 13	0.02
Male sex, n (%)	1,388 (76.8)	1,116 (77.4)	272 (74.3)	0.06	267 (75.9)	263 (74.7)	0.02
History of PCI, n (%)	179 (10.0)	140 (9.7)	39 (10.7)	-0.03	29 (8.2)	38 (10.8)	-0.07
History of CABG, n (%)	22 (1.2)	16 (1.1)	6 (1.6)	-0.04	4 (1.1)	6 (1.7)	-0.04
Prior myocardial infarction, n (%)	154 (8.6)	115 (8.1)	39 (10.7)	-0.08	33 (9.4)	39 (11.1)	-0.05
Prior heart failure, n (%)	62 (3.5)	48 (3.4)	14 (3.8)	-0.02	9 (2.6)	14 (4.0)	-0.07
Hypertension, n (%)	1,299 (75.7)	1,012 (74.3)	287 (81.1)	-0.15	290 (82.4)	286 (81.2)	0.02
Diabetes, n (%)	612 (35.7)	482 (35.4)	130 (36.7)	-0.04	124 (35.2)	130 (36.9)	-0.03
Dyslipidemia, n (%)	1,090 (63.5)	853 (62.6)	237 (66.9)	-0.09	232 (65.9)	237 (67.3)	-0.02
Smoking, n (%)	720 (42.0)	597 (43.8)	123 (34.7)	0.13	116 (33.0)	122 (34.7)	-0.03
Chronic kidney disease, n (%)	257 (15.0)	212 (15.6)	45 (12.7)	0.06	53 (15.1)	44 (12.5)	0.06
Dialysis, n (%)	36 (2.1)	25 (1.8)	11 (3.1)	-0.07	8 (2.3)	11 (3.1)	-0.04
Chronic obstructive lung disease, n (%)	39 (2.3)	34 (2.5)	5 (1.4)	0.06	4 (1.1)	5 (1.4)	-0.02
Peripheral artery disease, n (%)	56 (3.3)	49 (3.6)	7 (2.0)	0.07	11 (3.1)	7 (2.0)	0.06
Baseline hemoglobin (g/dL)	14.1 ± 2.1	14.1 ± 2.0	14.0 ± 2.1	0.00	14.0 ± 2.2	14.1 ± 2.2	-0.06
**Clinical presentations, n (%)**							
Cardiopulmonary arrest within 24 h	45 (2.5)	33 (2.3)	12 (3.3)	-0.05	4 (1.14)	11 (3.1)	-0.12
Cardiogenic shock within 24 h	59 (3.3)	49 (3.4)	10 (2.8)	0.03	8 (2.3)	10 (2.9)	-0.03
Acute heart failure within 24 h	68 (3.8)	57 (4.0)	11 (3.0)	0.04	18 (5.1)	11 (3.1)	0.08
Number of diseased vessels							
1	1,190 (65.9)	937 (65.0)	253 (69.1)	-0.07	228 (64.8)	243 (69.0)	-0.07
2	432 (23.9)	359 (24.9)	73 (19.9)	0.1	85 (24.1)	71 (20.2)	0.08
3	185 (10.2)	145 (10.1)	40 (10.9)	-0.02	39 (11.1)	38 (10.8)	0.01
Left main disease	10 (0.6)	9 (0.6)	1 (0.3)	0.04	2 (0.6)	1 (0.3)	0.03
**Preprocedural medications, n (%)**							
Antiplatelets	1,563 (86.5)	1,245 (86.4)	318 (86.9)	-0.01	313 (88.9)	307 (87.2)	0.04
Aspirin	1,531 (84.7)	1,218 (84.5)	313 (85.5)	-0.02	308 (87.5)	302 (85.8)	0.04
Clopidogrel	158 (8.7)	124 (8.6)	34 (9.3)	-0.02	28 (8.0)	32 (9.1)	-0.03

Prasugrel	1,296 (71.7)	1,039 (72.1)	257 (70.2)	0.03	267 (75.9)	248 (70.5)	0.1
Ticagrelor	1 (0.1)	1 (0.1)	0 (0)	0.03	0 (0)	0 (0)	NA
Oral anticoagulants	58 (3.2)	49 (3.4)	9 (2.5)	0.04	10 (2.8)	9 (2.6)	0.01
Warfarin	26 (1.4)	22 (1.5)	4 (1.1)	0.03	3 (0.9)	4 (1.1)	-0.02
Direct oral anticoagulant	34 (1.9)	29 (2.0)	5 (1.4)	0.04	10 (2.8)	5 (1.4)	0.08
**Preprocedural mechanical circulatory assist device, n (%)**							
ECMO	2 (0.1)	2 (0.1)	0 (0)	0.04	0 (0)	0 (0)	NA
Impella	1 (0.1)	1 (0.1)	0 (0)	0.03	0 (0)	0 (0)	NA
IABP	8 (0.4)	8 (0.4)	0 (0)	0.07	1 (0.3)	0 (0)	0.05
**Lesion locations, n (%)**							
Right coronary artery							
Segment 4	559 (30.9)	452 (31.4)	107 (29.2)	0.04	117 (33.2)	103 (29.3)	0.07
Left anterior descending artery							
Segment 8	113 (6.3)	94 (6.5)	19 (5.2)	0.05	19 (5.4)	19 (5.4)	0.00
Segment 9	469 (26.0)	315 (21.9)	154 (42.1)	-0.37	68 (19.3)	148 (42.0)	-0.43
Segment 10	34 (1.9)	22 (1.5)	12 (3.3)	-0.1	6 (1.7)	12 (3.4)	-0.09
Left circumflex artery							
Segment 12 or HL	304 (16.8)	265 (18.4)	39 (10.7)	0.17	66 (18.8)	36 (10.2)	0.19
Segment 14	181 (10.0)	161 (11.2)	20 (5.5)	0.16	40 (11.4)	19 (5.4)	0.17
Segment 15	147 (8.1)	132 (9.2)	15 (4.1)	0.16	36 (10.2)	15 (4.3)	0.18
**Devices used during PCI, n (%)**							
Rotational atherectomy	3 (0.2)	1 (0.07)	2 (0.5)	-0.08	0 (0)	2 (0.6)	-0.11
Thrombus aspiration	766 (42.4)	641 (44.5)	125 (34.2)	0.17	2 (0.6)	0 (0)	0.08
Distal protection device	14 (0.8)	14 (1.0)	0 (0)	0.1	2 (0.6)	0 (0)	0.08

Data are presented as n (%) or means (standard deviations).

CABG, coronary artery bypass graft; DCB, drug-coated balloon; DES, drug-eluting stent; eGFR, estimated glomerular filtration rate; ECMO, extracorporeal membrane oxygenation; HL, high lateral branch; IABP, intra-aortic balloon pumping; NA, not applicable; PCI, percutaneous coronary intervention; Std. diff, standardized difference.

Table S7. Baseline Characteristics Before and After Propensity Score Matching in NSTE-ACS in the Major Lesion Cohort.

	Non-matching				Matching		
	**All**	**DES**	**DCB**	**Std. diff**	**DES**	**DCB**	**Std. diff**
	**(n=17,045)**	**(n=16,073)**	**(n=972)**		**(n=910)**	**(n=910)**	
**Clinical characteristics**							
Age, years	70 ± 12	70 ± 12	70 ± 13	0.01	70 ± 13	70 ± 13	-0.01
Male sex, n (%)	12,775 (74.9)	12,073 (75.1)	702 (72.2)	0.05	664 (73.0)	660 (72.5)	0.01
History of PCI, n (%)	3,378 (19.9)	3,021 (18.8)	357 (36.8)	-0.35	332 (36.5)	337 (37.0)	-0.01
History of CABG, n (%)	403 (2.4)	359 (2.2)	44 (4.5)	-0.11	27 (3.0)	41 (4.5)	-0.07
Prior myocardial infarction, n (%)	2,098 (12.4)	1,913 (12.0)	185 (19.2)	-0.17	155 (17.0)	174 (19.1)	-0.04
Prior heart failure, n (%)	1,651 (9.8)	1,536 (9.6)	115 (12.0)	-0.06	101 (11.1)	112 (12.3)	-0.03
Hypertension, n (%)	12,496 (76.8)	11,760 (76.7)	736 (79.1)	-0.05	727 (79.9)	720 (79.1)	0.02
Diabetes, n (%)	6,376 (39.2)	5,972 (38.9)	404 (43.4)	-0.07	396 (43.5)	396 (43.5)	0.00
Dyslipidemia, n (%)	10,872 (66.8)	10,253 (66.8)	619 (66.6)	0.00	616 (67.7)	612 (67.3)	0.01
Smoking, n (%)	5,912 (36.3)	5,608 (36.6)	304 (32.7)	0.06	283 (31.1)	293 (32.2)	-0.02
Chronic kidney disease, n (%)	3,267 (20.1)	3,037 (19.8)	230 (24.7)	-0.1	223 (24.5)	217 (23.8)	0.01
Dialysis, n (%)	858 (5.3)	775 (5.1)	83 (8.9)	-0.13	84 (9.2)	79 (8.7)	0.02
Chronic obstructive lung disease, n (%)	472 (2.9)	447 (2.9)	25 (2.7)	0.01	11 (1.2)	25 (2.8)	-0.1
Peripheral artery disease, n (%)	841 (5.2)	779 (5.1)	62 (6.7)	-0.06	63 (6.9)	61 (6.7)	0.01
Baseline hemoglobin (g/dL)	13.4 ± 2.1	13.5 ± 2.1	13.0 ± 2.2	0.16	13.2 ± 2.2	13.1 ± 2.2	0.03
**Clinical presentations, n (%)**							
Cardiopulmonary arrest within 24 h	316 (1.9)	298 (1.9)	18 (1.9)	0.00	15 (1.7)	16 (1.8)	-0.01
Cardiogenic shock within 24 h	461 (2.7)	434 (2.7)	27 (2.8)	0.00	35 (3.9)	25 (2.8)	0.05
Acute heart failure within 24 h	804 (4.7)	759 (4.7)	45 (4.7)	0.00	42 (4.6)	40 (4.4)	0.01
Number of diseased vessels							
1	11,450 (67.2)	10,792 (67.1)	658 (67.7)	-0.01	617 (67.8)	611 (67.1)	0.01
2	3,766 (22.1)	3,566 (22.2)	200 (20.6)	0.03	201 (22.1)	189 (20.8)	0.03
3	1,829 (10.7)	1,715 (10.7)	114 (11.7)	-0.03	92 (10.1)	110 (12.1)	-0.05
Left main disease	489 (2.9)	471 (2.9)	18 (1.9)	0.06	32 (3.5)	17 (1.9)	0.08
**Preprocedural medications, n (%)**							
Antiplatelets	15,247 (89.5)	14,385 (89.5)	862 (88.7)	0.02	830 (91.2)	809 (88.9)	0.06
Aspirin	14,785 (96.8)	13,962 (96.9)	823 (95.4)	0.05	804 (96.6)	771 (95.2)	0.09
Clopidogrel	3,807 (24.9)	3,526 (24.5)	281 (32.6)	-0.13	247 (29.7)	268 (33.1)	-0.04

Prasugrel	10,049 (65.8)	9,556 (66.3)	493 (57.1)	0.14	498 (59.9)	463 (57.2)	0.06
Ticagrelor	25 (0.2)	24 (0.2)	1 (0.1)	0.01	1 (0.1)	1 (0.1)	0.00
Oral anticoagulants	1,008 (5.9)	948 (5.9)	60 (6.2)	-0.01	53 (5.8)	56 (6.2)	-0.01
Warfarin	357 (2.1)	337 (2.1)	20 (2.1)	0.00	21 (2.3)	18 (2.0)	0.02
Direct oral anticoagulant	691 (4.1)	651 (4.1)	40 (4.1)	0.00	36 (4.0)	38 (4.2)	-0.01
**Preprocedural mechanical circulatory assist device, n (%)**							
ECMO	33 (0.1)	31 (0.2)	2 (0.2)	0.00	0 (0)	2 (0.2)	-0.07
Impella	12 (0.1)	12 (0.1)	0 (0)	0.03	1 (0.1)	0 (0)	0.03
IABP	164 (1.0)	157 (1.0)	7 (0.7)	0.02	9 (1.0)	6 (0.7)	0.03
**Lesion locations, n (%)**							
Right coronary artery							
Segment 1	1,631 (9.6)	1,562 (9.72)	69 (7.1)	0.08	81 (8.9)	67 (7.4)	0.05
Segment 2	1,932 (11.3)	1,849 (11.5)	83 (8.5)	0.08	98 (10.8)	76 (8.4)	0.07
Segment 3	1,425 (8.4)	1,348 (8.4)	77 (7.9)	0.01	92 (10.1)	74 (8.1)	0.06
Left main trunk	522 (3.1)	506 (3.2)	16 (1.7)	0.08	35 (3.9)	14 (1.5)	0.11
Left anterior descending artery							
Segment 6	4,534 (26.6)	4,293 (26.7)	241 (24.8)	0.04	232 (25.5)	221 (24.3)	0.02
Segment 7	3,838 (22.5)	3,652 (22.7)	186 (19.1)	0.07	206 (22.6)	172 (18.9)	0.07
Left circumflex artery							
Segment 11	1,316 (7.7)	1,163 (7.2)	153 (15.7)	-0.23	79 (8.7)	147 (16.2)	-0.19
Segment 13	1,847 (10.8)	1,700 (10.6)	147 (15.1)	-0.11	87 (9.6)	139 (15.3)	-0.15
**Devices used during PCI, n (%)**							
Rotational atherectomy	463 (2.7)	390 (2.4)	73 (7.5)	-0.21	26 (2.9)	71 (7.8)	-0.2
Thrombus aspiration	2,074 (12.2)	1,988 (12.4)	86 (8.9)	0.09	84 (9.2)	79 (8.7)	0.02
Distal protection device	812 (4.8)	797 (5.0)	15 (1.5)	0.15	41 (4.5)	15 (1.7)	0.13

Data are presented as n (%) or means (standard deviations).

CABG, coronary artery bypass graft; DCB, drug-coated balloon; DES, drug-eluting stent; ECMO, extracorporeal membrane oxygenation; EGFR, estimated glomerular filtration rate; IABP, intra-aortic balloon pumping; NSTE-ACS, non-ST-elevation acute coronary syndrome; PCI, percutaneous coronary intervention; Std. diff, standardized difference.

Table S8. Baseline Characteristics Before and After Propensity Score Matching in NSTE-ACS in the Minor Lesion Cohort.

	Non-matching				Matching		
	All	DES	DCB	Std. diff	DES	DCB	Std. diff
	(n=2,801)	(n=2,048)	(n=753)		(n=717)	(n=717)	
**Clinical characteristics**							
Age, years	70 ± 12	70 ± 12	70 ± 12	-0.02	70 ± 12	70 ± 12	0.02
Male sex, n (%)	2,223 (79.4)	1,648 (80.5)	575 (76.4)	0.08	539 (75.2)	552 (77.0)	-0.03
History of PCI, n (%)	706 (25.3)	495 (24.3)	211 (28.0)	-0.07	194 (27.1)	203 (28.3)	-0.02
History of CABG, n (%)	73 (2.6)	47 (2.3)	26 (3.5)	-0.06	20 (2.8)	26 (3.6)	-0.04
Prior myocardial infarction, n (%)	397 (14.3)	276 (13.6)	121 (16.2)	-0.06	107 (14.9)	118 (16.5)	-0.03
Prior heart failure, n (%)	230 (8.3)	160 (7.9)	70 (9.3)	-0.04	58 (8.1)	66 (9.2)	-0.03
Hypertension, n (%)	2,163 (80.3)	1,590 (80.7)	573 (79.4)	0.03	577 (80.5)	568 (79.2)	0.03
Diabetes, n (%)	1,057 (39.2)	783 (39.7)	274 (38.0)	0.03	269 (37.5)	272 (37.9)	-0.01
Dyslipidemia, n (%)	1,850 (68.7)	1,343 (68.1)	507 (70.2)	-0.03	505 (70.4)	507 (70.7)	0.00
Smoking, n (%)	955 (35.5)	719 (36.5)	236 (32.7)	0.07	218 (30.4)	234 (32.6)	-0.04
Chronic kidney disease, n (%)	497 (18.5)	366 (18.6)	131 (18.1)	0.01	128 (17.9)	130 (18.1)	-0.01
Dialysis, n (%)	108 (4.0)	78 (4.0)	30 (4.2)	-0.01	20 (2.8)	29 (4.0)	-0.06
Chronic obstructive lung disease, n (%)	69 (2.6)	51 (2.6)	18 (2.5)	0.01	19 (2.7)	17 (2.4)	0.01
Peripheral artery disease, n (%)	130 (4.8)	96 (4.9)	34 (4.7)	0.01	34 (4.7)	34 (4.7)	0.00
Baseline hemoglobin (g/dL)	13.7 ± 2.1	13.7 ± 2.1	13.7 ± 2.1	0.00	13.7 ± 2.0	13.7 ± 2.1	0.00
**Clinical presentations, n (%)**							
Cardiopulmonary arrest within 24 h	25 (0.9)	23 (1.1)	2 (0.3)	0.08	11 (1.5)	2 (0.3)	0.1
Cardiogenic shock within 24 h	30 (1.1)	27 (1.3)	3 (0.4)	0.08	12 (1.7)	3 (0.4)	0.09
Acute heart failure within 24 h	57 (2.0)	46 (2.3)	11 (1.5)	0.05	19 (2.7)	10 (1.4)	0.07
Number of diseased vessels							
1	1,884 (67.3)	1,357 (66.3)	527 (70.0)	-0.07	502 (70.0)	505 (70.4)	-0.01
2	646 (23.1)	482 (23.5)	164 (21.8)	0.03	151 (21.1)	152 (21.2)	0.00
3	271 (9.7)	209 (10.2)	62 (8.2)	0.05	64 (8.9)	60 (8.4)	0.02
Left main disease	17 (0.6)	12 (0.6)	5 (0.7)	-0.01	2 (0.3)	5 (0.7)	-0.05
**Preprocedural medications, n (%)**							
Antiplatelets	2,488 (88.8)	1,824 (89.1)	664 (88.2)	0.02	641 (89.4)	635 (88.6)	0.02
Aspirin	2,431 (97.6)	1,785 (97.7)	646 (97.3)	0.03	624 (97.2)	621 (97.8)	0.01
Clopidogrel	598 (24.0)	432 (23.6)	166 (25.0)	-0.02	167 (26.0)	156 (24.6)	0.03

Prasugrel	1,632 (65.5)	1,216 (66.6)	416 (62.7)	0.07	409 (63.7)	402 (63.3)	0.02
Ticagrelor	0 (0)	0 (0)	0 (0)		0 (0)	0 (0)	NA
Oral anticoagulants	144 (5.1)	101 (4.9)	43 (5.7)	-0.03	40 (5.6)	42 (5.9)	-0.01
Warfarin	58 (2.1)	40 (2.0)	18 (2.4)	-0.02	12 (1.7)	18 (2.5)	-0.05
Direct oral anticoagulant	89 (3.3)	64 (3.1)	25 (3.3)	-0.01	28 (3.9)	24 (3.3)	0.02
**Preprocedural mechanical circulatory assist device, n (%)**							
ECMO	2 (0.1)	2 (0.1)	0 (0)	0.03	2 (0.3)	0 (0)	0.05
Impella	0 (0)	0 (0)	0 (0)	NA	0 (0)	0 (0)	NA
IABP	6 (0.2)	6 (0.3)	0 (0)	0.05	1 (0.1)	0 (0)	0.04
**Lesion locations, n (%)**							
Right coronary artery							
Segment 4	528 (18.9)	419 (20.1)	109 (14.5)	0.13	135 (18.8)	105 (14.6)	0.09
Left anterior descending artery							
Segment 8	140 (5.0)	111 (5.4)	29 (3.9)	0.06	45 (6.3)	28 (3.9)	0.09
Segment 9	689 (24.6)	388 (18.9)	301 (40.0)	-0.4	130 (18.1)	280 (39.1)	-0.4
Segment 10	60 (2.1)	29 (1.4)	31 (4.1)	-0.15	13 (1.8)	31 (4.3)	-0.13
Left circumflex artery							
Segment 12 or HL	781 (27.9)	620 (30.3)	161 (21.4)	0.16	232 (32.4)	153 (21.3)	0.2
Segment 14	401 (14.3)	319 (15.6)	82 (10.9)	0.11	112 (15.6)	81 (11.3)	0.1
Segment 15	202 (7.2)	162 (7.9)	40 (5.3)	0.08	50 (7.0)	39 (5.4)	0.05
**Devices used during PCI, n (%)**							
Rotational atherectomy	27 (1.0)	20 (1.0)	7 (0.9)	0.01	11 (1.5)	6 (0.8)	0.05
Thrombus aspiration	340 (12.1)	281 (13.7)	59 (7.8)	0.15	97 (13.5)	59 (8.2)	0.13
Distal protection device	15 (0.5)	13 (0.6)	2 (0.3)	0.04	4 (0.6)	2 (0.3)	0.03

Data are presented as n (%) or means (standard deviations).

CABG, coronary artery bypass graft; DCB, drug-coated balloon; DES, drug-eluting stent; eGFR, estimated glomerular filtration rate; ECMO, extracorporeal membrane oxygenation; HL, high lateral branch; IABP, intra-aortic balloon pumping; NA, not applicable; NSTE-ACS, non-ST-elevation acute coronary syndrome; PCI, percutaneous coronary intervention; Std. diff, standardized difference.

Table S9. Details of periprocedural complications.

	Overall				Major lesion cohort				Minor lesion cohort			
	DCB (n=2,606)	DES (n=2,606)	OR (95% CI)	P Value	DCB (n=1,537)	DES (n=1,537)	OR (95% CI)	P Value	DCB (n=1,069))	DES (n=1,069))	OR (95% CI)	P Value
Procedural myocardial infarction	6 (0.2)	9 (0.3)	0.67 (0.19-2.10)	0.61	4 (0.3)	7 (0.5)	0.57 (0.12-2.25)	0.55	2 (0.2)	0 (0)	NA	0.48
Cardiac tamponade	4 (0.2)	3 (0.1)	1.33 (0.23-9.11)	1.00	4 (0.3)	2 (0.1)	2.00 (0.29-22.16)	0.68	0 (0)	1 (0.1)	NA	1.00
Acute heart failure	15 (0.6)	27 (1.0)	0.55 (0.27-1.08)	0.09	13 (0.8)	21 (1.4)	0.62 (0.28-1.29)	0.23	2 (0.2)	2 (0.2)	0.62 (0.28-1.29)	1.00
Acute stent thrombosis	NA	4 (0.2)	NA	NA	NA	5 (0.3)	NA	NA	NA	0 (0)	NA	NA
Access site bleeding	1 (0.04)	7 (0.3)	0.14 (0.01-1.11)	0.08	0 (0)	5 (0.3)	NA	0.07	1 (0.1)	0 (0)	NA	1.00
Non-access site bleeding	1 (0.04)	5 (0.2)	0.20 (0.01-1.79)	0.22	1 (0.1)	4 (0.3)	0.25 (0.01-2.53)	0.37	0 (0)	0 (0)	NA	1.00
Emergency heart surgery	0 (0)	0 (0)	NA	NA	0 (0)	1 (0.1)	NA	1.00	0 (0)	0 (0)	NA	1.00

Data are presented as n (%). DCB, drug-coated balloon; DES, drug-eluting stent; OR, odds ratio; CI, confidence interval; NA, not applicable.

Figure S1. Illustration Defining Major and Minor Lesions in Coronary Arteries.

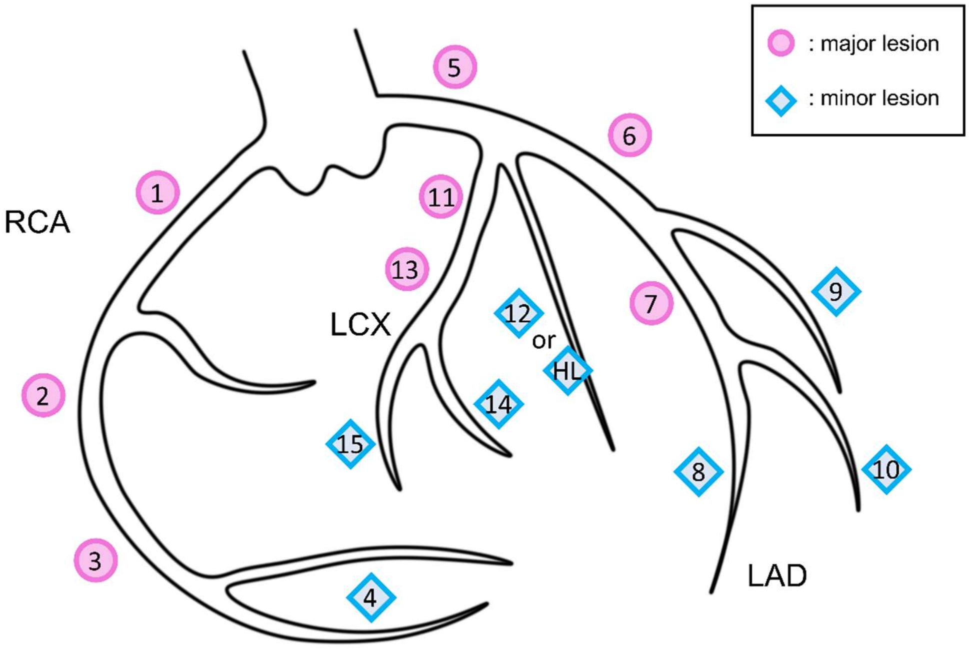

Light red circles represent major lesions, while light blue squares indicate minor lesions. The numbers inside the circles and squares represent the standard American Heart Association segmentation.

HL=high lateral branch.

Figure S2. Receiver Operating Characteristic Curves and Consistency of Propensity Score Matching.

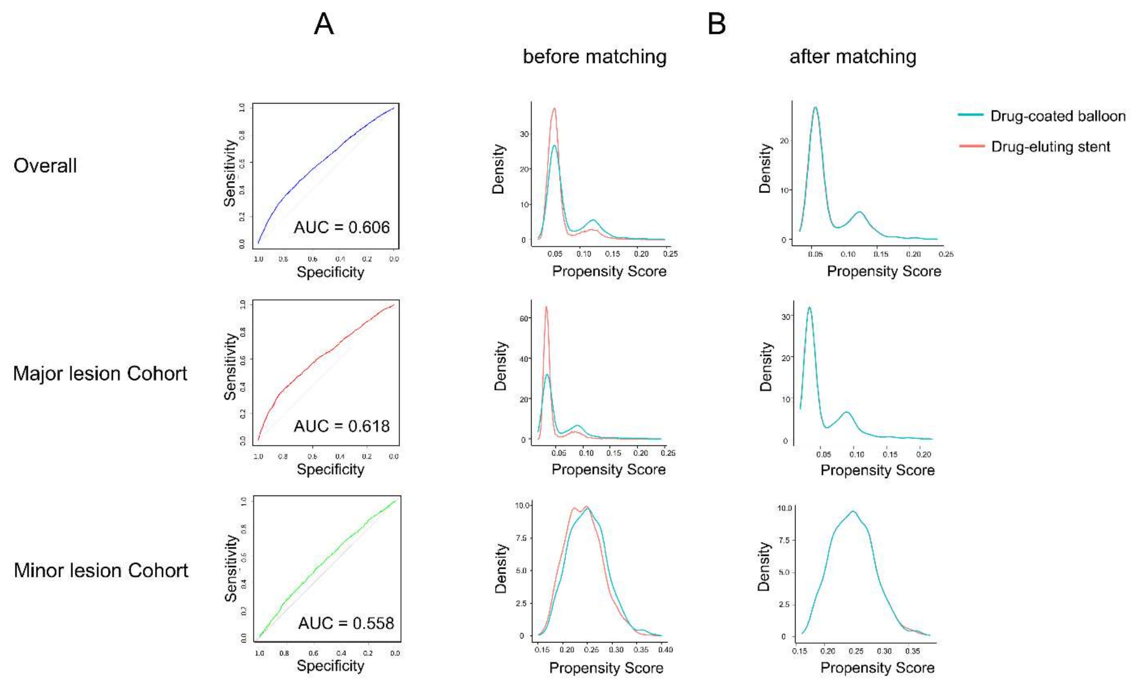

A) Receiver operating characteristic curves and concordance indices.

B) Comparison of the consistency of propensity score densities before and after matching.

AUC=area under the curve.
